# Summer warmth between 15,500 and 15,000 years ago enabled human repopulation of the northwest European margin

**DOI:** 10.1038/s41559-025-02712-9

**Published:** 2025-07-02

**Authors:** I. P. Matthews, A. P. Palmer, I. Candy, C. Francis, A. M. Abrook, P. C. Lincoln, S. P. E. Blockley, S. Engels, A. MacLeod, R. A. Staff, W. Z. Hoek, J. Burton

**Affiliations:** 1 Department of Geography, Royal Holloway, University of London, Egham, UK; 2 School of Geography and Environmental Science, University of Southampton, Southampton, UK; 3 School of Ocean and Earth Science, University of Southampton, Southampton, UK; 4 School of Social Sciences, Birkbeck, University of London, London, UK; 5 Department of Geography and Environmental Science, University of Reading, Reading, UK; 6 School of Archaeology, University of Oxford, Oxford, UK; 7 Department of Physical Geography, Faculty of Geosciences, Utrecht University, Utrecht, the Netherlands

**Keywords:** Palaeoclimate, Archaeology

## Abstract

High-magnitude decadal to centennial-scale abrupt changes in climate had a transformative effect on many past human populations. However, our understanding of these human/climate relationships is limited because robust tests of these linkages require region-specific quantified palaeoclimatic data with sufficient chronological precision to permit comparisons to the archaeological record. Here we present new high-resolution palaeoclimatic data and combine these with radiocarbon inventories of archaeological and faunal material, to test the relationship between abrupt warming and the ability of humans to rapidly repopulate the northwest margins of Europe (>50° N and encompassing the area of Britain, Ireland, the surrounding islands and the North Sea basin) after regional abandonment during the Last Glacial Maximum. We address the timing of this process and the relevance of the abrupt climate changes recorded in the Greenland ice cores. We use the IntCal20 radiocarbon calibration curve to show that the earliest human repopulation in this region occurred up to 500 years before the climate of Greenland warmed. However, our analyses show that parts of the northwest European margin had already experienced substantial summer warming by this time, probably driven by changes of sea-ice area in the eastern North Atlantic. The associated warming influenced the distribution of key hunter-gatherer prey species such as reindeer, which were a key resource for humans. Accordingly, this study highlights asynchrony in seasonal warming across the North Atlantic region during the last deglaciation and shows that this asynchrony permitted human exploitation of northwest European margin paraglacial landscapes by ~15,200 years before the present.

## Abstract (web summary)

The authors use the latest radiocarbon calibration curve and new local palaeoclimatic and palaeoenvironmental records from Britain to show that humans repopulated the northwest European margin between 15,500 and 15,000 years ago, supported by local summer warmth occurring earlier than previously thought.

## Main

The last deglaciation of the major Northern Hemisphere ice sheets began between 24 and 18 thousand years ago (ka)^1,2^, during a period of cool, unstable climates terminating in abrupt warming at ~14.64 ka BP, the onset of the Lateglacial Interstadial (or Bølling in Europe)^3^. This amelioration is clearly recorded and chronologically defined by abrupt warming to interstadial conditions in the Greenland ice cores that began at 14.64 ± 0.19 (maximum counting error) GICC05 ka BP (Greenland Interstadial 1 (GI-1))^4^. The driver of the dispersal of early humans from centres in southwestern France and northern Spain into more northern areas, which were previously uninhabited during the Last Glacial Maximum (LGM), is much debated^5–7^. However, it is believed that after 18 ka, an initial dispersal began from southern refugia, which then spread into some areas of northern Europe possibly as early as 17 ka^8^. For Britain, Ireland and the surrounding North Sea basin at the northwest (NW) European margin, this dispersal occurred in closer association with the time of the onset of warm GI-1 conditions^9,10^.

Most studies considering the climatic context of human repopulation during deglaciation rely, in part, on comparison to the climate record of the Greenland ice cores^9,11–13^. The δ^18^O of the ice cores is considered a proxy for palaeotemperature that is supported by a high-precision chronology generated across multiple Greenland ice-core records^3^. Furthermore, the climate event stratigraphy constructed from these archives is the established high-resolution chrono-stratotype for climatic transitions of this period^3^. Despite these attributes, any archaeological study that uses the Greenland ice cores as a climatic framework to understand human dispersal or activity must make three major assumptions, which are frequently unproven: first, that the abrupt climate transitions defined in the Greenland ice cores are clearly represented in the regions where human dispersal is being investigated; second, that the timing of climatic transitions in the study area is synchronous with the timing of those transitions in the ice cores; and third, that palaeotemperatures recorded in Greenland accurately reflect the prevailing environmental conditions encountered by humans in areas away from Greenland^14^. These underlying assumptions are untenable, as existing terrestrial palaeoclimate data show that the Greenland record does not contain all abrupt climatic events through the Last Glacial–Interglacial Transition (LGIT) and that the timing of these events may not be synchronous across the Northern Hemisphere^15–17^. These issues restrict our understanding of how Palaeolithic populations responded to rapidly changing and spatially heterogeneous glacial climates.

The repopulation on the margins of NW Europe after the last deglaciation exemplifies the difficulties in testing past human–climate interactions. Numerous archaeological sites dating to the Lateglacial Interstadial support the idea that the warm, temperate climates during this interval aided the dispersal of humans into the region^18^. However, for archaeological sites in central and eastern Europe, there is strong evidence that repopulation occurred earlier, before or around 17 ka, meaning that the relationship between climate, environment and archaeological response is uncertain^8,19–25^. In contrast, the data for the NW European margin is more closely associated with and tested against warming in Greenland, leading to uncertainty over drivers and responses to these climatic transitions across the continent^7,9,12,26–29^. Much of the uncertainty in the order of climatic and repopulation events probably reflects the continued reporting of older, less reliable ^14^C dates, often prepared without the most reliable pretreatment methods^30,31^ and calibrated using older radiocarbon calibration curves^32–34^. It is feasible that any apparent discrepancy in the timing of human dispersal and climatic change may reflect inaccurate archaeological dating or underlying issues with the radiocarbon calibration curve^7,35,36^. Using the most refined radiocarbon methods, Jacobi and Higham generated dates related to human repopulation of the European NW margin^18^. These dates suggested that this dispersal of humans occurred 200–400 years prior to the onset of GI-1, although the chronological uncertainties associated with radiocarbon calibration at the time of publication meant that some chronological overlap with the GI-1 warming could not be excluded. Consequently, an untested discrepancy exists where humans seemingly repopulated the NW European margin prior to a climatic shift that is supposed to have driven the dispersal. It is currently unclear (1) whether humans had indeed dispersed into the NW European margin before the high-magnitude and abrupt GI-1 warming and, consequently, whether the timing of human migration was regulated not by climate but rather by societal adaptation as seen elsewhere in Europe^37^; (2) whether the northerly latitudes around the current British land mass experienced some warming prior to 14.64 cal. ka BP, allowing humans to repopulate this region in advance of the larger-scale climatic warming event in Greenland; or (3) whether there is an issue in the comparison of timescales underlying the climatic and archaeological data due to offsets between the Greenland ice-core and the radiocarbon (IntCal) timescales^38^.

**Fig. 1:**
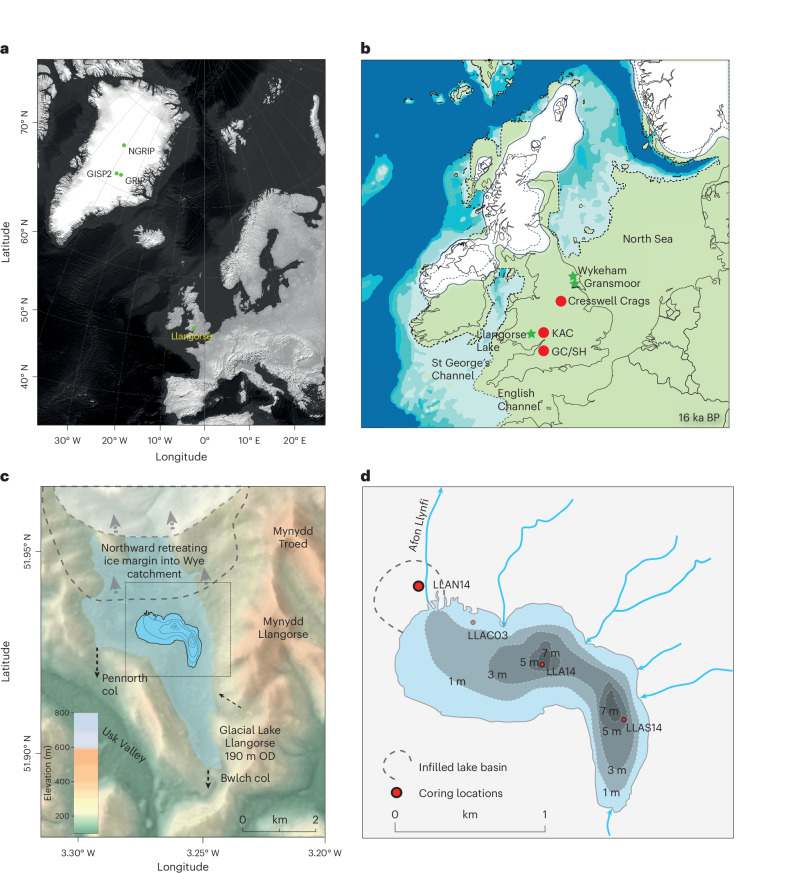
Location maps of the sites mentioned in the text. **a**, Location of the Llangorse basin in Europe and Greenland ice-core sites. Data from ref. ^108^. **b**, Llangorse Lake and key Late Palaeolithic sites and other palaeoenvironmental records referred to in the text within the British land mass (green) and the ice sheet extent (white) at 16 ka (ref. ^2^). **c**, Topographic setting of the lake basin, which emerged after the retreat from the local LGM. **d**, The current lake basin, its bathymetry and the location of the LLAN14 core in the infilled basin. Basemaps in **a** and **d** from ref. ^50^ under a Creative Commons license CC BY 4.0; topography in **c** from ref. ^109^ under a Creative Commons license CC BY 4.0.

Here we address the first two questions by presenting new evidence from southern Britain that allows us to establish the palaeoclimatic and palaeoenvironmental conditions that existed during the repopulation of the NW European margin. We address the third by combining these data with radiocarbon-dated archaeological remains for Britain recalibrated using the IntCal20 calibration curve^38,39^ and harmonized with Greenland climate records by rescaling the chronology of the Greenland isotope data to IntCal20. This rescaling was accomplished by matching periodic short-term solar modulations recorded by ^10^Be flux in Greenland to δ^14^C estimates inferred from IntCal20 by Engels et al.^40,41^. Our new palaeoclimatic record from Llangorse, South Wales (Fig. 1), is located near the earliest British archaeological sites, contains an expanded sediment stratigraphy of the period and is supported by a high-resolution chronology based on an inventory of 27 radiocarbon dates showing that sedimentation occurred across the full deglacial period. Stable oxygen isotope (δ^18^O_bulk_) and chironomid analysis (C-IT_Jul_) are used to quantify the climatic conditions during deglaciation and climatic warming. Palaeoenvironmental responses to the identified climatic variations are derived from pollen and sedimentological data, enabling the construction of a model of broader landscape responses to climatic change into which humans were migrating at this time. These data are then compared to the Greenland palaeoclimatic data, key regional climatic proxy records, radiocarbon-dated faunal data and TraCE-21ka model outputs of sea-ice extent from across the North Atlantic to understand both the climatic and environmental drivers of repopulation of northern Europe.

## Results and discussion

**Fig. 2:**
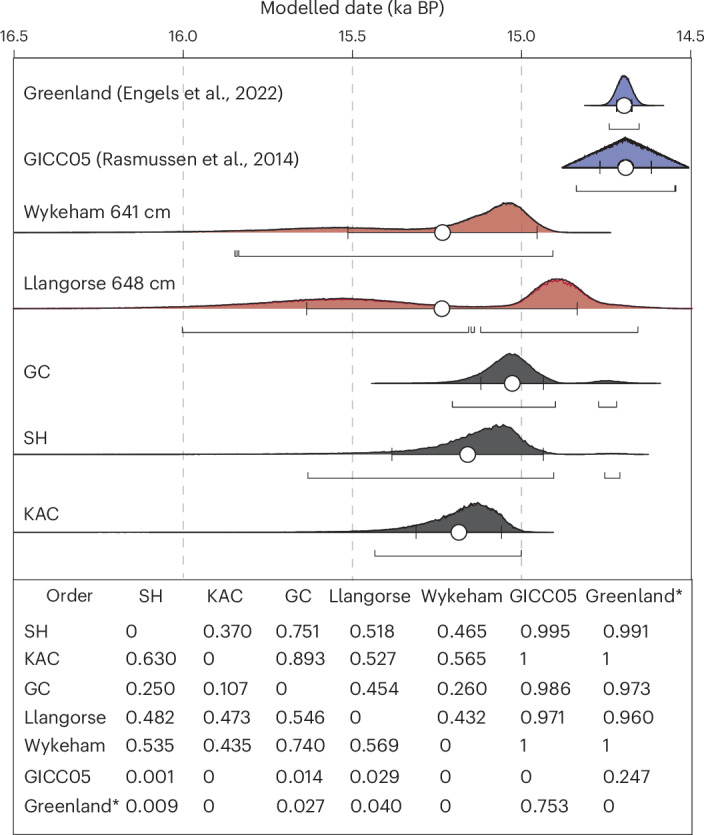
The timing of warming across the NW European margin compared with the archaeological data. Age estimates for the onset of warming in the Llangorse Lake record (648.5 cm; Extended Data Fig. 2; red), remodelled age distributions for the onset of the warming of the palaeoclimate record from Wykeham (red), human reoccupation at selected sites in the British land mass (black) and oxygen isotope record from the Greenland ice core during the LGIT (blue). Two age distributions are provided for the onset of the Greenland GI-1e warming using (1) the GICC05 estimate^3^ and (2) the conversion to IntCal20 years BP (ref. ^40^); the latter is marked by an asterisk^40^. The black age distributions are remodelled radiocarbon ages for archaeological material recovered from specific sites in the British land mass. The circles and error bars represent mean values and one sigma ranges, while the bars below indicate the modelled 95.4% probability ranges. The table at the bottom represents a matrix of probabilities of the order of events. High values (>0.9) indicate high likelihood that the data/site represented in the row precedes the data/event in the column. The results suggest that all archaeological sites included here have a high probability of activity before warming in Greenland (>0.986 probability for GICC05 and >0.973 for the Engels et al.^40^ age model), while both NW European margin palaeoclimatic proxies indicate a high probability of warming before Greenland (>0.960 for Llangorse and 1.0 for Wykeham).

**Extended Data Fig. 1:**
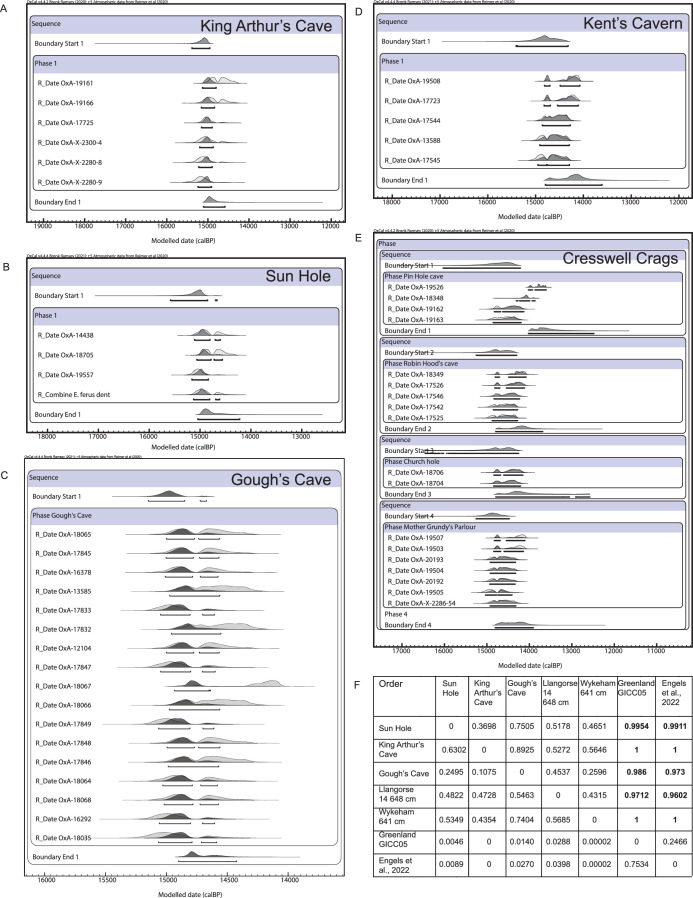
OxCal Phase models for selected cave sites in the British Isles. **A**) King Arthur’s Cave with the dates reported by Jacobi and Higham (2011) and were on human-modified horse bone. The onset of the Phase pre-dates warming in Greenland. **B**) Sun Hole, Cheddar Gorge Somerset. The dates were reported by Jacobi and Higham (2011) and were on humanly modified horse bone and human bone (OxA-19557). The onset of the Phase largely pre-dates warming in Greenland with some overlap in the youngest part of the distribution. The individual date on the human bone pre-dates warming in Greenland when adjusted to the IntCal20 timescale. **C**) Gough’s Cave, Cheddar Gorge Somerset. The dates were reported by Jacobi and Higham (2011) and were on horse, deer, dog, reindeer, lynx and human bone, with one date from mammoth ivory. The dates on human bone (OxA-17847 – OxA-17849) coincide with the onset of warming in Greenland. **D**) Kents Cavern, Torquay, Devon. The dates were reported by Jacobi and Higham (2011) and were on horse, dog, and bovid. **E**) Cresswell Crags, Derbyshire. The dates were reported by Jacobi and Higham (2011) and were on horse and Arctic hare. Unlike Jacobi and Higham (2011) the dates are placed in Phases by excavation location rather than species. As remarked by Jacobi and Higham (2011) the phases of activity occur later than those seen in the Cheddar locations and at King Arthur’s Cave. **F**) Order of event probabilities for human occupation in archaeological locations and the onset of warming in the key palaeoclimatic archives discussed in the text. The probability is that t1 > t2.

Evidence for the repopulation of the NW European margin during the last deglaciation is based on the identification and dating of stone tools, skeletal remains and human-modified bone^42^. In Britain, the earliest evidence for the repopulation is referred to as the (Final) Magdalenian or Creswellian phase^43,44^. To establish the best constrained date of human reoccupation of Britain, we have taken the radiocarbon dates on ultrafiltered human-modified animal bones or human remains from the earliest ‘Creswellian’ British sites reported in refs. ^18,45^ and recalibrated these dates using IntCal20 within site-based Bayesian phase models (EDF 1)^18,39,46,47^. The oldest evidence for repopulation of the NW margin of Europe comes from King Arthur’s Cave (KAC), which shows a phase of activity beginning between 15.2 and 15.0 cal. ka BP. The recalibrated dates from KAC show that human occupation of southern Britain occurred 300–500 years prior to warming in Greenland (probability 1.0; Fig. 2 and Extended Data Fig. 1). The sites of Sun Hole (SH) and Gough’s Cave (GC) show evidence for local human occupation slightly post-dating the phase of activity at KAC, but both SH and GC have high probabilities (0.99 and 0.97, respectively) of human activity pre-dating the warming in Greenland (Fig. 2). The comparison of recalibrated dates derived from existing archaeological evidence to the Greenland ice-core records synchronized to the IntCal20 timescale clearly shows that the observed differences in timing are not driven by chronological uncertainties but indicative of a centennial-scale offset between human occupation of the NW European margin and warmer temperatures in Greenland.

### Palaeoenvironmental results

**Extended Data Fig. 2:**
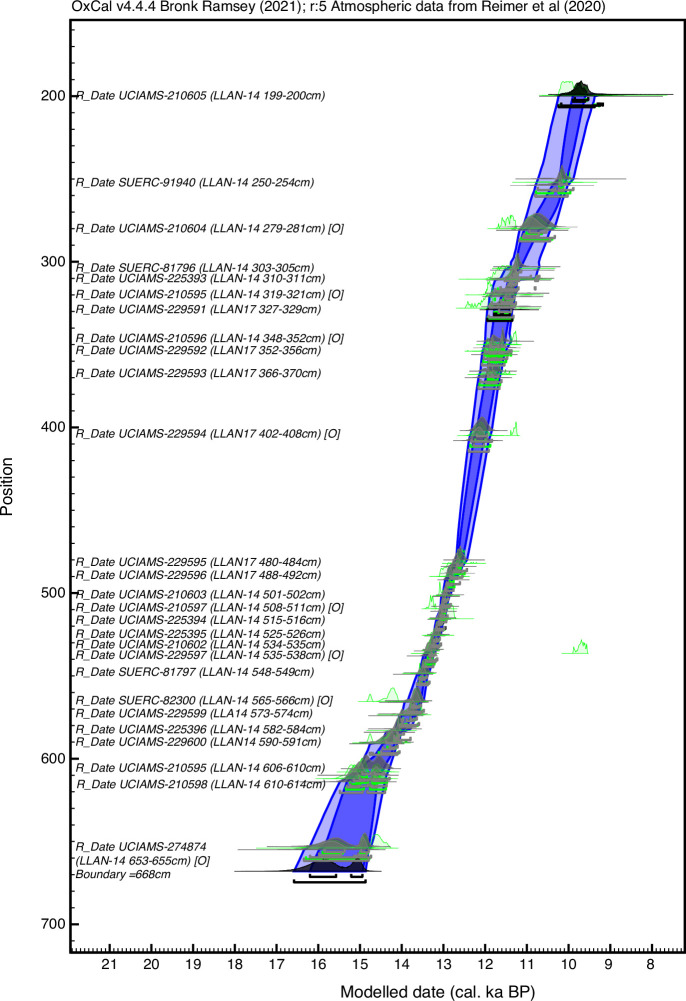
The Age-depth model for Llangorse. The final Age-depth model for Llangorse, outlier dates (>50%) are marked by an O. Dark blue shading denotes 68.3% range and light blue indicates the 95.4% range.

To examine potential climatic asynchrony between the NW European margin and Greenland, a palaeoclimatic record with independent dates was generated from Llangorse Lake, South Wales. The Llangorse record is unusual in the NW European margin as it spans the LGIT from the period of local deglaciation at ~20–18 ka ^48–52^ to the present. A Bayesian-age model was constructed using 27 radiocarbon measurements from terrestrial plant macrofossil remains, which demonstrates continuous high-resolution sedimentation between ~15.20 and 9.68 cal. ka BP (Extended Data Fig. 2).

**Fig. 3:**
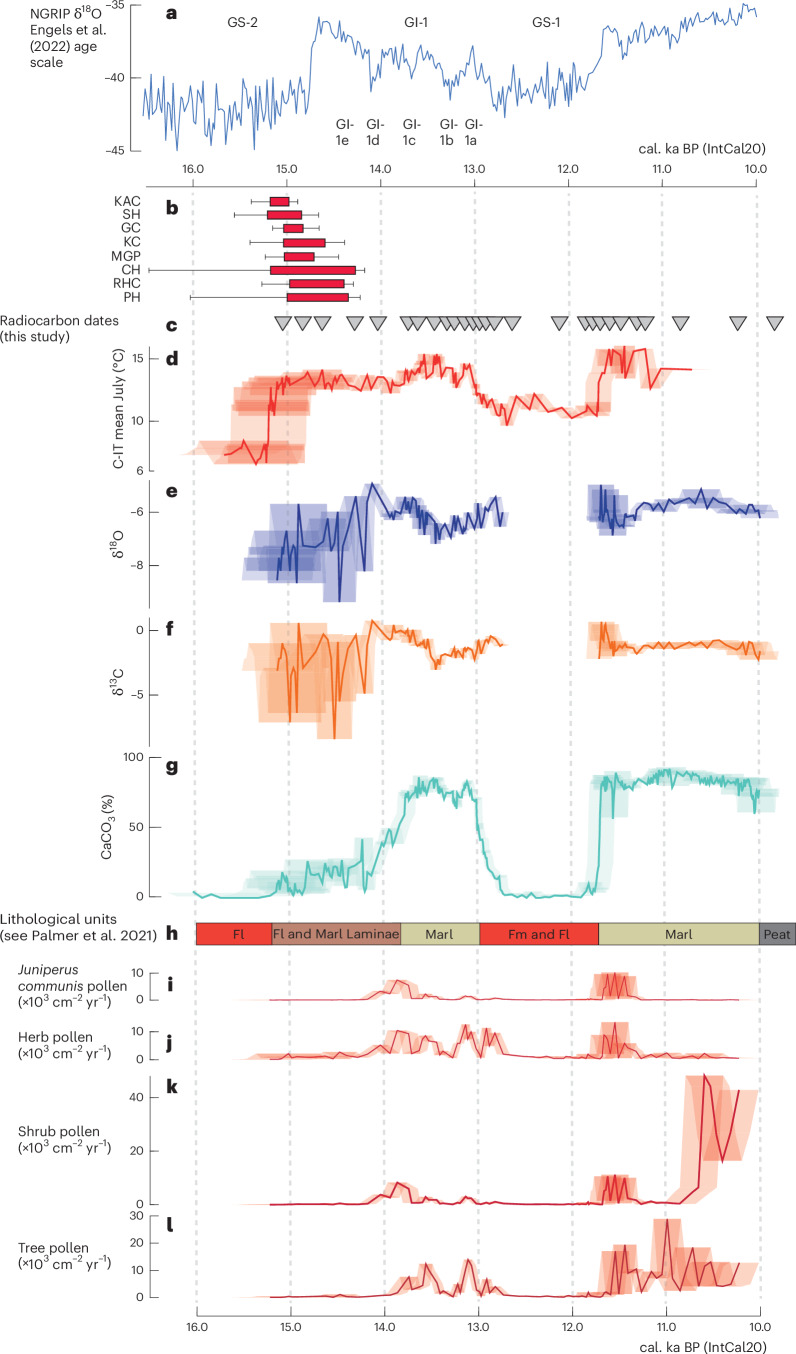
Comparing the archaeological data to the Llangorse record. **a**–**l**, Comparison of the British archaeological dataset (**b**) to the Greenland warming recorded in the oxygen isotope record (**a**) and the palaeoclimatic and palaeoenvironmental proxy data from Llangorse (LLAN14, **d**–**l**). Archaeological dates are represented by red boxes (68% probability range) with black bars (95.4% probability range). The palaeoclimatic proxy data from Llangorse include chironomid-inferred mean July temperatures (**d**) (Extended Data Fig. 3) and stable isotope ratios of oxygen (**e**) and carbon (**f**) (Extended Data Fig. 4), while environmental changes are presented using sediment CaCO_3_ (**g**). The main litholological units in LLAN14 are provided for context (**h**), and vegetation reconstructions using pollen accumulation rate calculations for *Juniperus* (**i**), total trees (**j**), shrubs (**k**) and herbs (**l**) are shown (Extended Data Figure 5). In **d**–**g** and **i**–**l**, the error envelopes indicate the 95.4% range from the age model. Inverted triangles indicate the positions of the 27 radiocarbon dates used to construct the age model for the palaeoclimatic and palaeoenvironmental datasets (**c**) (Extended Data Fig. 2). KC, Kent’s Cavern; MGP, Mother Grundy’s Parlour; CH, Church Hole; RHC, Robin Hood’s Cave; PH, Pin Hole.

The sediment sequence records the major climatic intervals of the LGIT with the deposition of CaCO_3_-rich marls reflecting warm intervals (that is, parts of the Lateglacial Interstadial and the early Holocene), while the deposition of mineral-rich silts and clays mostly reflects the cold intervals at the end of the LGM and the Lateglacial Stadial (~Younger Dryas; Fig. 3). This interpretation is supported by our temperature reconstruction, which indicates that higher temperatures are broadly coincident with increased per cent CaCO_3_, while low temperatures correspond to periods of decreased per cent CaCO_3_ (Fig. 3). Anomalous to this general trend is the period between 15.20 and 14.00 cal. ka BP, where CaCO_3_ percentages are relatively low (~5–50%), but during which July temperatures increased to between 12 and 14 °C. The presence of mineral-dominated deposits alongside warm conditions is probably caused by the characteristic delay in landscape and sediment response to climatic amelioration^53,54^. This means that, although temperatures may have increased, a time lag of up to thousands of years exists for vegetation to become established and surface processes to stabilize, particularly after a climatic deterioration as substantial as the LGM^50,55,56^. Indeed, the mineral-dominated sediments in the lower part of the core are characterized by very thin laminae of marl, suggesting a complex sedimentological response to what appear to be warmer climatic conditions^38^ (Fig. 2).

**Extended Data Fig. 3:**
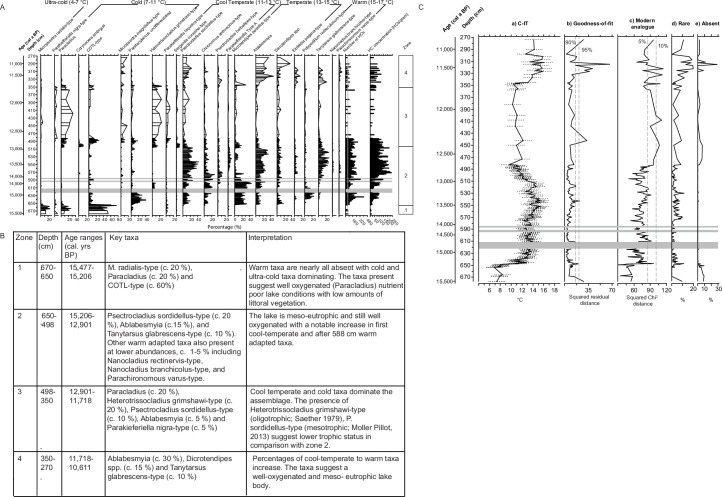
Chironomid data from Llangorse. **A**) Percentage abundance diagram of selected chironomid taxa present in the LLAN14 sequence from Llangorse. Taxa are arranged by their T_Jul_ optima in the modern calibration dataset (Heiri et al.^83^) as calculated using a Weighted-Averaging model. Head capsule concentrations (HCs/gram) and total head capsules counts per sample are displayed on the right. Grey shading denotes samples which have been removed due to no or very few HCs. **B**) Chironomid zone descriptions. **C**) Chironomid numerical data. **a**) Chironomid-inferred mean July air temperature estimates (T_Jul_) with sample specific error bars; **b**) Goodness-of-fit of the fossil assemblages to temperature, with dashed lines representing the 90^th^ and 95^th^ percentile of squared residual distance of modern samples to the first axis of a CCA; **c**) Nearest modern analogue with dashed lines showing the 5^th^ and 10^th^ percentile of the Chi^2^ distances of all samples in the modern calibration dataset. Grey shading denotes samples which have been removed due to no or very few head capsules. Ten samples fall below the generally acceptable threshold of 50 head capsules. There is a good fit to temperature in 148 samples (88%) with a further 12 having a ‘poor fit’ and another 8 with a ‘very poor fit’ to temperature. Crucially, apart from the very basal sample no ‘very poor fit to temperature samples’ occur in the basal sediments (700 – 600 cm) which define the warming. Two samples in the interval 700 – 600 cm have poor analogues (tenth percentile) in the training set with no samples in this interval have very poor analogues (fifth percentile). Overall, we take these outputs as evidence that temperature is the primary driving influence on chironomid ecology except for a short interval in the Early Holocene sediments.

The Llangorse record has two key palaeoclimate proxy records (C-IT_Jul_ and δ^18^O_bulk_). The high-resolution chironomid record of Llangorse (Fig. 3 and Extended Data Fig. 3) shows shifts between assemblages dominated by cold-indicating taxa and assemblages characterized by warm-indicating taxa (Fig. 3 and Extended Data Fig. 5). Mean July air temperatures were reconstructed from the subfossil chironomid dataset using a chironomid–climate calibration dataset. C-IT_Jul_ had exceeded 10–12 °C by ~15.20 ka BP, and high values were maintained in the range of 12–14 °C until ~13.80 ka BP. Between ~13.80 and ~13.00 ka BP, temperatures ranged from 14 to 16 °C before declining to 9–12 °C in the subsequent stadial (~Younger Dryas). Modern July temperatures for South Wales are ~16 °C, and the C-IT_Jul_ record from Llangorse therefore shows that summer temperature anomalies were as little as 2 °C below modern levels by 15.20 cal. ka BP and modern values attained between 13.80 and 13.00 cal. ka BP. This study suggests there is a high probability (0.96) that warming at Llangorse started ~500 years prior to abrupt warming in Greenland and that the initial phase of higher temperatures (12–14 °C) in Llangorse was established ~400 years earlier (Fig. 2) than in Greenland, which was still recording cold conditions associated with the later part of Heinrich Stadial 1 (Fig. 3).

**Extended Data Fig. 4:**
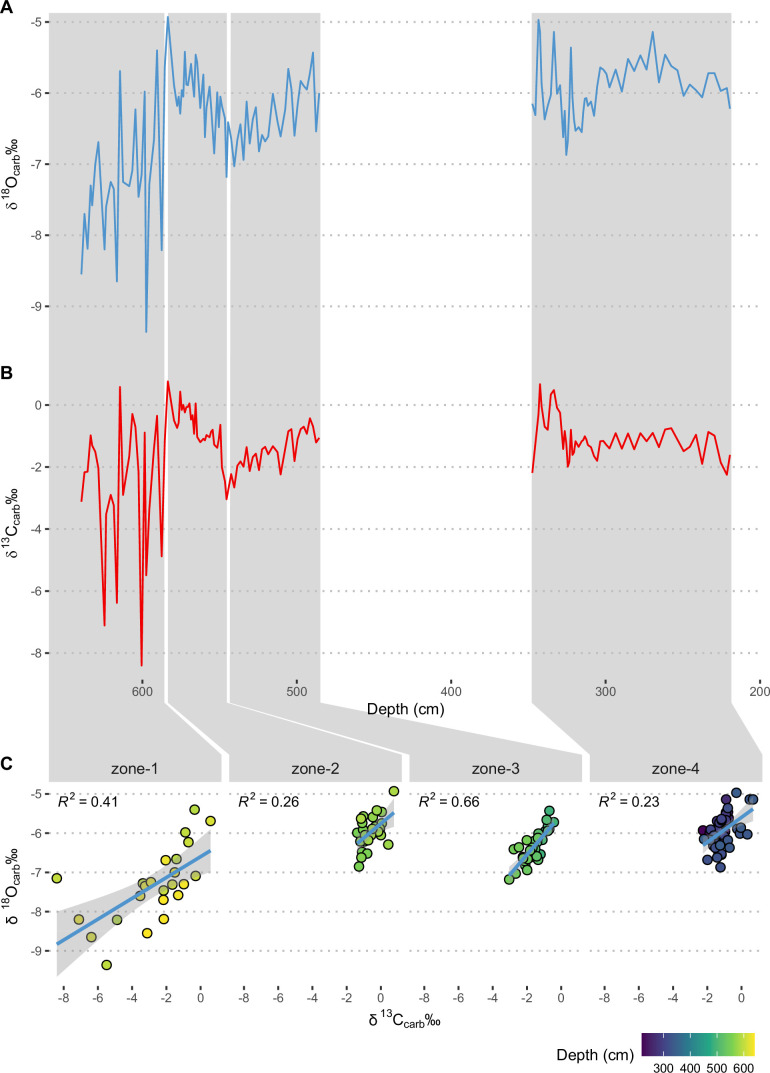
Isotopic covariance plots for the Llangorse sediments. **A** = down core δ^18^O variation, **B** = down core δ^13^C variation, **C** = Oxygen and carbon isotopic covariance across four zones during the Lateglacial. Each scatter plot includes a linear regression model fit with standard error and r^2^ value. Zone 1 dates to the period ca 15.20 - 14.16 ka, zone 2 to between 14.16 – 13.51 ka, zone 3 to 13.51 – 12.74 ka, and zone 4 to the Holocene <11.70 ka BP. Higher covariance values (r^2^ = 0.66) can only be measured in zone 3 after 13.51 ka BP.

This pattern of climate variability is supported by the δ^18^O record from the CaCO_3_-rich sediments (Fig. 3). The δ^18^O record of lake carbonates in the Lateglacial of NW Europe is frequently used as a proxy for temperatures^16,17,57–61^ (Extended Data Fig. 4). This inference is based on two key assumptions: first, that the δ^18^O of lake carbonates is strongly controlled by the δ^18^O of the lake water, which, in the absence of major isotopic modification of the lake water through processes such as evaporation, is controlled by the δ^18^O of precipitation^58,62,63^; and, second, that the δ^18^O of precipitation is closely linked to air temperature^16,62,63^. This occurs either through the direct effect of air temperature on isotopic fractionation during condensation and precipitation, with colder air temperatures producing lower δ^18^O values, or through changes in air mass source/trajectory with the δ^18^O of moisture from colder northerly sources being more negative than that in warmer sources^62^. Many researchers have highlighted that the close agreement between changes, across the Lateglacial, in lake carbonate δ^18^O values and independent temperature estimates supports this interpretation^16,17,58,62–65^.

Our results are consistent with this interpretation and with previous studies, as a comparison between the Llangorse δ^18^O and C-IT_Jul_ records for the cooler interval between 15.20 and 14.10 cal. ka BP (C-IT_Jul_ of 12–14 °C) shows that it is characterized by lower δ^18^O values than those that occurred during the warmer interval between 14.10 and 13.51 cal. ka BP (C-IT_Jul_ of 14–16 °C). During the later part of the interstadial period, there is also an increasing degree of covariance between δ^18^O and δ^13^C that is interpreted as a sign of evaporation, indicating that after 13.51 cal. ka BP the isotopic record is related more closely to a humidity/aridity signal rather than purely to temperature (Extended Data Fig. 4). During this interval of increasing covariance, the positive linear relationship between C-IT_Jul_ and δ^18^O values breaks down, supporting the suggestion that isotopic values at the end of the interstadial are less closely related to temperature. This isotopic covariance is, however, absent from most of the interstadial section, suggesting that the δ^18^O signal during the earlier part of the interstadial record is not strongly affected by evaporation. If the isotopic signal of this part of the record is driven by temperature, then it is notable that the δ^18^O values are, for the most part, slightly lower than early Holocene δ^18^O values. This would suggest, in agreement with the C-IT_Jul_ values, an overall temperature regime slightly cooler than that of the current interglacial. However, during a few intervals the δ^18^O values are as high as those between 11.00 and 10.50 cal. ka BP, thereby suggesting that periods of warmth between 15.20 and 13.90 cal. ka BP were comparable to those of the early Holocene.

**Fig. 4:**
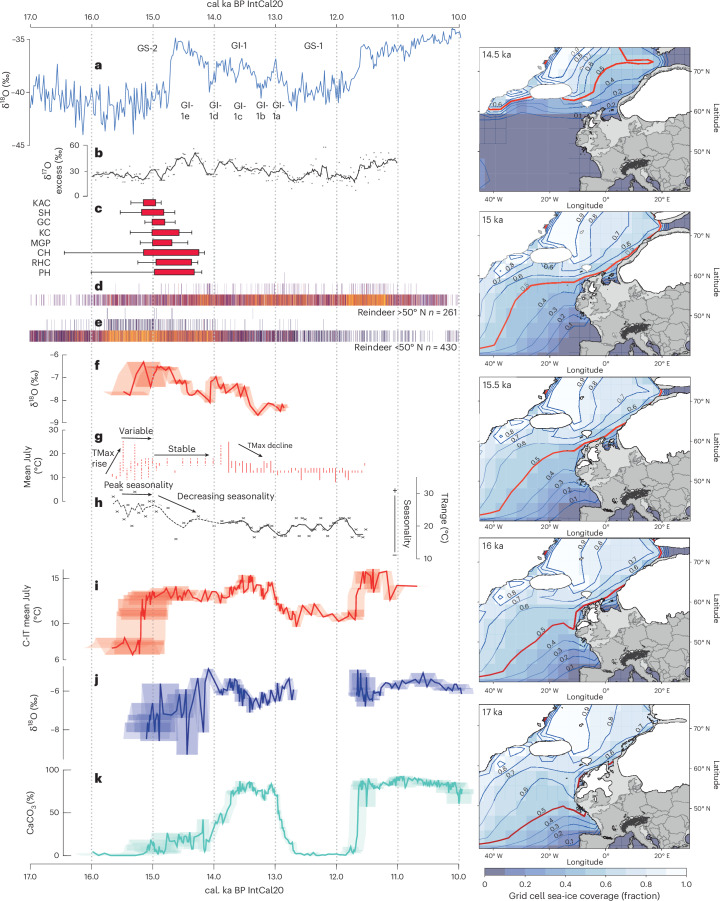
Comparison of environmental and climate records after 16 ka from the NW European margins with the Greenland ice-core record and modelled sea-ice extent. Comparison of environmental and climate records after 16 ka from the NW European margins with the Greenland ice-core record (**a**,**b**), evidence for earliest human occupation in the British land mass (**c**) and western European reindeer presence and absence (**d**,**e**). Panels **a** and **b** show the Greenland ice-core stable isotope (**a**) (with the age model adjusted using Engels et al.^40^) and ^17^O excess record (**b**)^73^ after 16 ka BP. Panel **c** shows the remodelled ages of evidence for human reoccupation in the British land mass (Fig. 3 and Extended Data Fig. 1). Red boxes represent 68% probability range and black bars represent 95.4% probability range. Western European reindeer presence and absence north (**d**) and south of 50° N (**e**) are presented using the RECE approach (Extended Data Fig. 7). **f**–**h**, Quantified palaeotemperature reconstruction from North Yorkshire (Gransmoor) from beetle mutual climatic ranges for mean July temperatures (**g**) and seasonal temperature range (TRange) (**h**)^57,67^ and inferred enhanced temperature from oxygen isotopic analyses at Wykeham^57^ (**f**), which indicate warming on the British land mass prior to the onset of GI-1e in Greenland. **i**–**k**, Selected palaeoenvironmental and palaeoclimatic proxies from Llangorse (LLAN14) including chironomid-inferred temperature (**i**), oxygen isotope (**j**) and calcium carbonate content (**k**) proxies are presented. In **f** and **i**–**k**, the error envelopes indicate the 95.4% range from the age model. The right panels show the output from the TraCE-21ka climate model to reconstruct the percentage sea-ice coverage of grid cells across the North Atlantic at time intervals between 17.00 ka and 14.50 ka. The red line marks the contour position where 50% of the grid cell is covered by sea ice in a year.

The evidence from the δ^18^O signal and the C-IT_Jul_ record show that summer warming had begun in southern Britain by ~15.20 cal. ka BP, ~500 years before the onset of GI-1. This centennial-scale offset suggests climatic asynchrony between Greenland and Britain during this period. The possibility of warming prior to 14.64 cal. ka BP in the NW European margins has been suggested previously^14,57,66–68^ (Fig. 4 and Extended Data Fig. 6). However, the chronological imprecision and/or the low resolution of the proxy data available in many of the sites has meant that establishing the precise timing of the onset of warming was not possible. By contrast, several European alpine records show warming dated several centuries after that observed at Llangorse^69,70^. A later warming detected in these European records could have been caused by delayed lake sediment accumulation arising from persistent permafrost in the catchments^54^, proxies responding to climatic variables other than summer temperature^71^, lack of chronological control on the timing of these events and/or genuine asynchrony within the climate across Europe^72^.

The suggestion that warm mean summer temperatures (~12–14 °C) were established in the NW European margin by 15.20 cal. ka BP is further supported by two lines of evidence: first from the animal bones identified in the archaeological sites listed above, and second in nearby palaeolake archives. At KAC, stable isotopic analysis of *Equus ferus* tooth material recovered from sediments linked to earliest human occupation (~15.14 ± 0.13 cal. ka BP) indicate warming from LGM conditions with mean annual temperatures approaching but still lower than present-day conditions^10^. In addition, two palaeolake archives in Northern England (Wykeham and Gransmoor, Yorkshire) have palaeoclimatic proxy data that suggest early warming (Figs. 2 and 4). At Wykeham, a period of oxygen isotopic enrichment interpreted as an increase in temperatures is observed and dated to 15.18 ± 0.28 cal. ka BP (Figs. 2 and 4 and Extended Data Fig. 7)^57^. This age estimate places warming at Wykeham earlier than in Greenland (probability 1.0, Fig. 2), but most likely contemporaneous with Llangorse. At Gransmoor, the timing of warming is more difficult to establish due to lower available chronological precision, but a period of warmer conditions^67^ is found to have been established prior to the earliest datable horizon at ~14.64 cal. ka BP^7^. Llangorse, Wykeham and Gransmoor register no further summer warming coincident with Greenland at 14.64 ka. Therefore, the palaeoclimate records derived from these sites on the NW European margin might contradict the consensus of European climatic change archives during the Lateglacial. However, we suggest that our findings do not preclude additional climatic adjustments across other parts of Europe or around the NW European margin coincident with warming in Greenland. The apparent anomaly could be explained by spatiotemporal variability across Europe producing seasonal changes of different magnitudes. The climate proxies at Llangorse responded to changes in summer temperature, while at Gransmoor the reconstructions suggest that cool winter conditions continued until ~14.64 cal. ka BP when warming occurred in winter^67^. Additionally, there is likely evidence of a time transgressive shift in warming that impacts different parts of the European mainland, which might be further complicated by proxy sensitivity to climatic forcing in different parts of Europe and/or seasonality. Nonetheless, there is an emerging picture of earlier, warmer summer temperatures around the NW European margin.

### Possible drivers of warming at the NW European margin

We compared empirical data and climate model information to understand what conditions drove asynchronous summer temperature trends between Greenland and Britain and how this facilitated repopulation. We have developed a model involving the localized retreat of summer sea ice from the coast of the British land mass and the NW European seaboard, but with continued sea-ice presence in the wider North Atlantic (Fig. 4 and Methods). This situation would allow the terrestrial margins of NW Europe to undergo warming, due to the increased influence of warmer ocean currents, but simultaneously would result in areas such as Greenland remaining cold. The likelihood of such a scenario can be assessed through regional proxies for atmospheric circulation and existing climate model data. δ^17^O excess measurements from NGRIP^73^ provide a proxy for the source water location of Greenland precipitation^73^. While temperatures over Greenland remain depressed until 14.64 cal. ka BP, δ^17^O excess values show a distinct increase from ~15.15 ka (Fig. 4) that can be attributed to a restricted northward shift in the source water area. Such a shift is best accounted for by a northward retreat in sea-ice extent at lower latitudes, including the area around Britain, while Greenland itself is still surrounded by sea ice until a further, and much larger, δ^17^O excess increase at ~14.64 ka BP concomitant with the abrupt warming seen in the ice cores.

Climate model simulations using the TraCE-21ka climate model support these inferences. The simulations show that grid cells with at least 50% sea-ice cover, which we use to represent the position of dominant ice cover in the North Atlantic, were identifiable off the west coast of France with sea-ice cover greater than 50% at latitudes >50° N at 17.0 ka (Fig. 4 and Methods). However, between 16 and 15.5 ka the model suggests that dominant sea-ice cover on the NW European margin had moved northward to approximately 55–60° N but remained static at approximately <42° N in the mid-Atlantic. The transition to more open water in the eastern Atlantic permitted warmer water and possibly Atlantic air masses to extend into the NW European margins. A final northward shift in annual sea-ice extent to the southern margins of Iceland (>60° N) occurred between 15.0 and 14.5 ka, with much of the North Atlantic remaining open during the summer months. This pattern of sea-ice retreat would permit at least seasonal summer warming earlier in the latitudes of the NW European margins around Llangorse before 15.2 ka. Crucially, it would enable later warming to be observed in the higher latitudes around Greenland.

### Drivers of repopulation of the NW European margin

**Extended Data Fig. 5:**
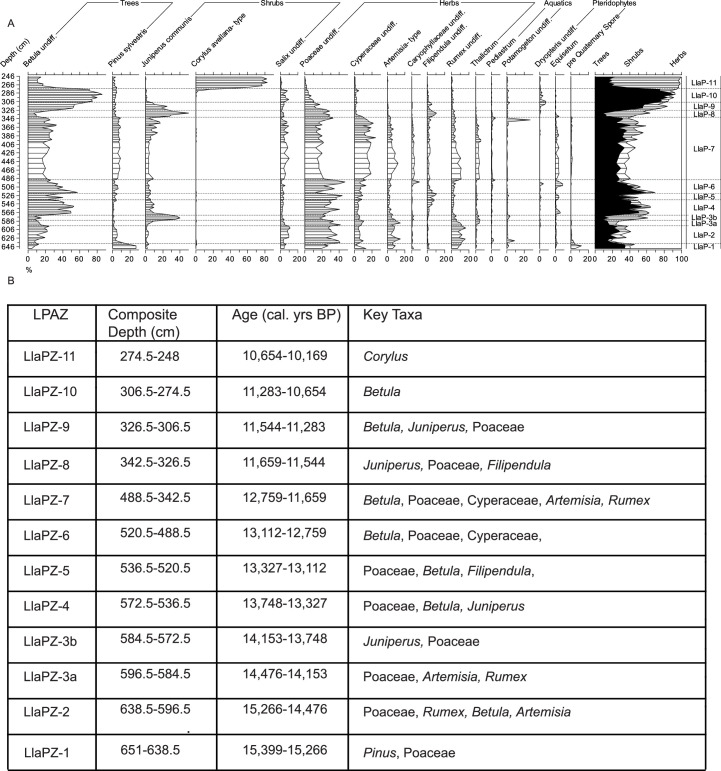
Pollen data for Llangorse. **A**) Summary Percentage Pollen data for Llangorse lake. **B**) Pollen zones and key taxa for LLAN 14.

**Extended Data Fig. 6:**
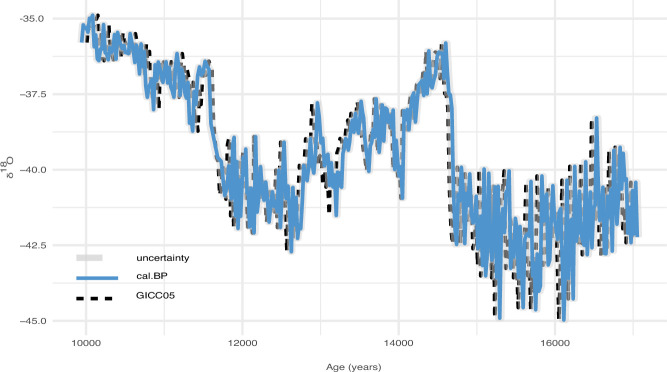
Greenland δ18O record rescaled to the IntCal20 timescale. Greenland δ^18^O record rescaled to IntCal20 including matching the uncertainty as reported by Engels et al.^40^.

For hunter-gatherer populations to disperse successfully and repopulate a region to the level of archaeological visibility, the landscape needs more than warmer climatic conditions; it must also be able to support a prey-species population. Although seasonally warm climates persisted from ~15.20 cal. ka onwards, the landscape and ecology around Llangorse remained in a typical glacial/paraglacial state, suggesting that neither the temperature records at Llangorse nor those from Greenland are good measures of the local environmental conditions at this time. The prevalence of minerogenic sedimentation in the lake until 13.80 cal. ka BP indicates that the lake was surrounded by unstable slopes and soils vulnerable to high erosion rates and degradation, while the pollen data suggest extremely low levels of vegetation cover dominated by arctic/alpine herb and shrub taxa, with thermophiles (for example, *Juniperus*^66^) being present only after 14.40 cal. ka BP (Extended Data Fig. 5). This paraglacial landscape is a legacy of the preceding glaciation, and, despite the warming summer temperatures, the development of thermophilous plant taxa in this landscape is delayed by ~1,000 years and the appearance of tree taxa by ~1,400 years. Consequently, a distinct climatic and ecological niche consisting of warm summer climates with grasses and most probably moss-dominated ecosystems and highly unstable landscapes existed, and it was into these marginal ecological niches that human dispersal occurred.

To test whether prey species migration might explain the early repopulation of northern Europe, we took a radiocarbon-dated event count ensemble (RECE) approach for all dated evidence of reindeer and horse, both high-ranked prey species in the Upper Palaeolithic period^74,75^. The available data for this exercise were not ultrafiltered, unlike the radiocarbon dates reported for the archaeological sites above. The reindeer and horse dates may lack the precision and accuracy of ultrafiltered dating, and therefore caveats must be placed on their interpretation; but this is still considered a useful way of determining faunal movements at multi-centennial scales. Here we present the analysis of the larger reindeer dataset, while the horse data and analysis are presented in Extended Data Fig. 8. Following previous research^76^, we divided western Europe into two sectors (south and north of 50° N) and calculated the RECE for the entire dataset and for these two sectors separately (Fig. 4). The results suggest increased probabilities of reindeer presence in NW Europe >50° N from ~16.00 cal. ka BP, with higher levels of visibility and a more consistent presence from ~15.00 cal. ka BP (Fig. 4). We suggest that the increase in the presence of reindeer reflects an expansion of their range (and presumably those of other large herbivores such as horse) at latitudes >50° N. We further suggest that this range expansion was driven by the marginal changes in summer warmth at Llangorse and other sites in the region, and this provided a food source for humans.

## Conclusion

Our new results from Llangorse strongly suggest that seasonal climate warming driven by spatial variability in sea-ice extent facilitated the repopulation of the NW European margin during the last deglaciation by 15.20 cal. ka BP. The rapidity of this repopulation is stark, with evidence for human presence in Britain coincident with local warming, even when the uncertainties of the respective chronologies are considered. This suggests that the climate shift was a prerequisite for repopulation, while our analyses show that the warming led to the northward migration of key hunter-gatherer prey species such as reindeer. Previous suggestions of the arrival of humans prior to climatic warming relied on correlation with internationally important but geographically distant climate records, an approach that we show requires too many assumptions to be relied on. Therefore, the construction of high-resolution palaeoclimatic and environmental records that are close to key archaeological sites is clearly imperative when dealing with complex climatic intervals, such as the last deglaciation, when divergent regional climates may have frequently occurred. Only if this can be achieved can the complex relationship between human populations and climate forcing be more robustly established.

## Methods

### Radiocarbon dating and Bayesian age modelling

Llangorse sediment was sampled at 1-cm intervals and sieved using deionized water over a 200-µm mesh. Terrestrial plant macrofossil material was extracted using a low-powered, stereozoom microscope. Where plant macrofossil material was present only in low concentrations, adjacent 1-cm samples were combined to provide sufficient material to generate a robust radiocarbon date. A standard acid–base–acid pretreatment protocol was applied at the Scottish Universities Environmental Research Centre, and subsequent accelerator mass spectrometry measurements were performed at both the Scottish Universities Environmental Research Centre and the University of California, Irvine, accelerator mass spectrometry facilities. The radiocarbon determinations and calibrated ages obtained using the IntCal20 calibration curve^39^ are presented in Supplementary Table 1.

Bayesian age–depth models were produced for both Llangorse and Wykeham using the OxCal v.4.4 software^47^. A P_Sequence deposition model was applied, implementing a variable *k* parameter to allow the program to independently derive the optimal rigidity of the age–depth relationship produced^47,77^. To accommodate the depth ranges of individual samples (≤6 cm, though predominantly ≤3 cm), a series of independent Sequences were constructed, consisting of the radiocarbon determinations themselves placed between Boundaries demarking the top and bottom of each sampling depth. These Boundaries were then cross-referenced into the principal P_Sequence model, following the methodology of refs. ^57,78^. Outlier analysis was additionally applied to objectively down-weight the influence of inconsistent data points using the General outlier model and giving each sample a prior probability of 5% of being an outlier^47^. The resultant age–depth model is shown in Extended Data Fig. 2. Of the 27 radiocarbon determinations included in the Llangorse model, eight were identified as having ≥50% posterior probability of being an outlier, perhaps relating to the difficulty inherent in dating small samples.

### Recalibration of radiocarbon dates and age modelling of material from British Upper Palaeolithic sites

The radiocarbon data presented by Jacobi and Higham^18^ were remodelled in OxCal v.4.4 using the IntCal20 calibration curve. Each site was included in an OxCal Phase model, and the likely onset of each phase was extracted for comparison with the Llangorse data. The approach mirrors that of the original publication, and the results are presented in Extended Data Fig. 1 and discussed further in the Supplementary Information.

### Rescaling the Greenland ice-core chronology to IntCal20

We followed the approach of Engels et al.^40^, using their code to provide a scaled record of oxygen isotope variability for the Lateglacial period and specifically across the onset of warming at GI-1e. The total range of the originally reported scaling covered the period 15–11.4 ka BP. No scaling was available for 15–17 ka or after 11 ka, and therefore the offsets identified at the oldest and youngest parts of the Engels et al.^40^ timescale are propagated back to 17 ka and forward to 10 ka. This scaling probably does not reflect the true offsets for this period, but all data sit within the maximum counting uncertainty of the Greenland record, therefore providing a robust measure of dating uncertainty.

### Resolving an order of events

Once the Greenland warming had been placed on a comparable timescale to the data from Llangorse and other chronologies, the age distributions for the archaeological phases and climatic warming event were extracted. The age probability distributions for the onsets of activity at the three oldest archaeological sites (KAC, SH and GC) were also collated. The output compared the three archaeological sites against the GICC05 age for warming, the Engels et al.^40^ scaled age for warming in Greenland, the onset of summer warming at Llangorse and warming at Wykeham. These data were extracted as priors and then placed within an OxCal v.4.3 Order command, which calculates a matrix of probabilities of the likelihood of age distributions preceding or overlapping one another. For Wykeham, the data reported by Lincoln et al.^57^ were recalibrated, and the probability distribution of the age at the interval 641 cm was extracted (Extended Data Fig. 8). This depth was selected because it represented enriched δ^18^O values interpreted as climatic amelioration, and the depth 638–641 cm also represents the sediment intervals for the lowermost ^14^C date in the sequence.

### Llangorse palaeoclimatic and palaeoenvironmental proxy data

The coring and analytical strategy within the Llangorse basin is described in Palmer et al.^50^. The sediment core analysed here is LLAN1, which was recovered from the northern margins of the former lake basin, which has been infilled subsequently. The core was obtained using a 1-m-long Russian corer with a 5-cm diameter. Calcium carbonate analysis was conducted using the Bascomb calcimeter method^79^. Radiocarbon samples were extracted from cores aligned on the basis of the lithological units from the same coring expedition in 2014. Where additional material was required, parallel cores were recovered and linked to the original sequence using 1-cm-interval CaCO_3_ records.

### Llangorse chironomid analysis

We extracted 168 chironomid samples through the LGIT section of LLAN14. The standard method^80^ did not adequately disaggregate the marl and clay-rich sediments^80^. We therefore took additional steps, including pre-picking the samples before placing them in an ultrasonic bath. The samples were then re-sieved to remove the newly disaggregated sediment to allow remaining head capsules (HCs) to be picked^81^. In general, HC preservation was good, although *Paratanytarsus austriacus*-type and *Paratanytarsus penicillatus*-type had to be combined in the fossil assemblages and the calibration dataset (*Paratanytarsus* undifferentiated), as morphotypes could not be differentiated due to missing mouthparts. *Corynocera oliveri*-type and *Tanytarsus lugens*-type were also combined (*COTL*-type), as they were difficult to distinguish.

A minimum count sum of 50 was targeted for each sample. We were unable to reach this amount in 14 samples due to low HC concentrations. Samples with <50 HCs were all located between 613 and 622 cm core depth. In the lower part of the sequence, adjacent samples between 633 and 639 cm core depth were combined to 2-cm intervals to reach a minimum of 50 HCs. Samples at 622 cm, 612 cm, 608 cm, 593 cm, 592 cm, 587 cm, 586 cm and 585 cm were left out of the final dataset as they could not be combined with adjacent samples to reach a count sum that would allow numerical analysis^82^. Qualitative assessments of the small number of taxa identified in these regions suggest coherence with the taxa found immediately above and below these sections. The final dataset used for numerical analysis, including temperature inference, included 157 samples.

### Chironomid-based temperature reconstruction

As can be seen in the above, temperature is considered to have been the most important driver of chironomid assemblage composition across the LGIT at Llangorse. We therefore applied a chironomid–climate inference model to the fossil record to produce a quantitative reconstruction of past July air temperatures (*T*_Jul_). We selected the 274-lake combined Swiss–Norwegian chironomid–climate calibration dataset, excluding 13 outlier lakes, as our preferred calibration dataset^82,83^. Unlike the Norwegian chironomid–climate calibration dataset, which is often applied to LGIT chironomid records from the UK^84,85^, the Swiss–Norwegian calibration dataset includes lakes with similar alkaline chemistry to Llangorse, contains a greater number of chironomid taxa and generates a longer temperature gradient between 3.5 and 18.4 °C. A two-component weighted averaging partial least squares regression model (Extended Data Fig. 3) was selected as the basis for our reconstructions, as this model combines a root mean squared error of prediction of 1.4 °C, with a coefficient of determination (*r*^2^_boot_) of 0.9 and a maximum bias of 0.8 °C (refs. ^86,87^). Sample-specific errors were calculated using 999 bootstrapping cycles^88^. Weighted averaging partial least squares regression analysis was performed in C2 (ref. ^89^).

The reliability of the *T*_Jul_ reconstruction was assessed using analogue and goodness-of-fit tests. Goodness-of-fit to temperature tests were performed by passively plotting fossil samples on the first axis of a canonical correspondence analysis (CCA) of the modern calibration dataset, using *T*_Jul_ as the sole constraining variable^88^. Fossil samples with a squared residual distance value that exceeded the 90th or 95th percentile of distances of samples in the modern calibration dataset to the first CCA axis were classified as having a ‘poor’ or ‘very poor’ fit to temperature, respectively^88^. CCA was conducted in R using the vegan package^90^. The majority of chironomid samples have good fit to temperature (Extended Data Fig. 3). Of the 157 chironomid samples, only 8 have a ‘very poor’ fit to temperature, and 12 have a ‘poor’ fit to temperature. All of these, except the sample at 677 cm, are from the Holocene section of the sequence.

Modern analogue technique analyses were performed to assess the similarity of fossil assemblages to those in the modern calibration dataset using the analogue package in R^91^. Squared-chord distance was used as a measure of dissimilarity, and samples were not square-root transformed prior to analysis. Fossil samples with distances to the closest modern analogue larger than the 5th or 10th percentile of all modern distances were treated as having ‘no close’ or ‘no good’ analogue, respectively^88,91^. The fossil assemblages compare well with those in the modern calibration dataset (Extended Data Figure 3c), with only 7 samples having ‘very poor’ analogues and 25 having ‘poor’ analogues, most of which are in zones 3 and 4 (Loch Lomond Stadial and the Holocene).

The majority of taxa present are well represented in the modern calibration dataset (Extended Data Fig. 3). Taxa that are rare in the modern dataset (that is, Hill’s N2 < 5) generally remain below 10% of the combined abundance for the sequence and do not form a major proportion of the assemblages, except in the early Holocene, where total per cent abundances can increase to ~20%. There are only three taxa that are not present in the modern-day calibration dataset but were present in the sediment sequence: *Constempellina-Thienemanniola*, *Trissocladius* and *Psilopserus*. These taxa are present in only 35 samples and generally form less than 10% of the assemblages, except in one sample in the early Holocene (315 cm) in which 29% of HCs are from taxa that are absent from the modern calibration dataset.

### Determination of the onset of warming

We defined the onset of mean July warming within the chironomid temperature reconstruction using a Bayesian changepoint analysis using 100,000 simulations with a fixed number of 20,000 burn-in iterations to determine the optimal number and positions of significant variations in temperature. This analysis was performed with the PAST computer program v.4.09 (ref. ^92^) using the method of Gallagher et al.^93^. The results define the first warming in the Llangorse data as occurring at the most probable depth of 648.5 cm. This may be a conservative estimate as temperatures begin to trend upward from 650 cm. The age for the 648.5-cm interval was extracted from the age–depth model.

### Llangorse stable isotope analysis

We extracted 138 isotope samples through LLAN14 with a 1-cm sample resolution. The samples were disaggregated in 0.5% sodium hexametaphosphate and sieved through a 63-µm mesh to remove ostracod and mollusc shell fragments and plant macrofossil material. The <63-µm fraction was immersed in 5% hydrogen peroxide to digest any finer organic material until the reaction stopped. The samples were subsequently rinsed and centrifuged three times, and then left to air dry. Stable isotope analysis was conducted in two laboratories, the Department of Earth Sciences at Royal Holloway University of London and the Department of Earth Sciences at University College London. At Royal Holloway University of London, a Cahn C-31 Microbalance was used to weigh the sample before measurements were performed using a VG PRISM series 2 mass spectrometer. Internal (RHBNC-PRISM) and external (NBS-19, LSVEC) standards were run every ten samples. At University College London, a Mettler Toledo XP6 microbalance and a ThermoFisher Delta Plus XP mass spectrometer with a Gasbench II preparation system were used with internal standards (BDH) analysed every three samples and three external standards (NBS-19) run prior to every machine run. In both laboratories, the samples were digested using phosphoric acid at 90 °C to evolve CO_2_. The results are presented with reference to Vienna Pee Dee Belemnite.

### Llangorse pollen analysis

The composite LLAN14 profile was sampled for pollen at variable resolutions, resulting in 88 samples. Between 655.5 and 486.5 cm and 402.5 and 248 cm, sediments were analysed at 4-cm resolution, while between 486.5 and 402.5 cm, sediments were sampled at a lower 12-cm resolution. Two different preparation procedures were applied to samples obtained from the Llangorse sequence. Principally, cubic centimetres of sediment were processed following standard procedures^94,95^, including the addition of *Lycopodium* to enable the estimation of pollen concentrations and pollen accumulation rates. The samples were treated with hydrochloric acid followed by a density separation approach, using sodium polytungstate at a specific gravity of 2.0 g cm^−3^, to separate the organic and clastic materials. The samples were subsequently acetolysed following Erdtman’s acetolysis^96^. Owing to a conspicuous lack of palynological material in the lowermost samples, between 655.5 and 594.5 cm, the sediments were resampled and processed using hydrofluoric acid^95^. Hydrofluoric acid treatment was performed after the hydrochloric acid stage but prior to acetolysis. Both methods of preparation were combined to enable a complete palynological sample set. The resultant palynomorph materials from both procedures were mounted using glycerine jelly.

Pollen identification was performed using an Olympus CX-41 binocular microscope at ×400 magnification with critical identifications conducted at ×1,000 magnification using immersion oil. Pollen identification was supported by pollen compendia with nomenclature updated to reflect recent botanical taxonomy^95,97,98^. Minimum count sums of 300 total land pollen (TLP) were obtained for all samples except for 650.5 cm, 644.5 cm and 640.5 cm, where a lack of pollen meant that sums of 100 TLP were deemed sufficient. Land pollen percentages were constructed as a function of TLP, with aquatic and pteridophyte percentages calculated using TLP and aquatics and TLP and pteridophytes, respectively^99^ (Extended Data Fig. 5). In the main paper, we present pollen accumulation rate data, calculated according to the method used by Engels et al.^40^.

### TraCE-21ka model simulation

Simulations of sea-ice extent were obtained from the global, coupled ocean–atmosphere–sea-ice–land-surface climate model simulation TraCE-21ka (https://www.earthsystemgrid.org/project/trace.html). TraCE-21ka uses the Community Climate System Model version 3, forced by orbitally driven insolation and greenhouse gas concentrations, and transient boundary conditions including the extent and topography of ice sheets, and changes in sea level. TraCE-21ka also prescribes a transient scenario of freshwater forcing into the oceans from deglaciating ice sheets. The sea-ice model employed in TraCE-21ka is the dynamic-thermodynamic National Center for Atmospheric Research Community Sea Ice Model with a longitudinal resolution of 3.6° and a variable latitudinal resolution, with finer resolution near the equator (~0.9°). The TraCE-21ka simulations have been shown to replicate the main features in global hydroclimatic reconstructions over the past 21,000 years, including the major shifts in Greenland temperatures over the last deglaciation.

We calculated 100-year means of seasonal and mean annual sea-ice extent from the full TraCE-21ka simulation (with transient forcing changes in greenhouse gases, orbitally driven insolation variation, ice sheets and meltwater fluxes) using the climate data operators command line suite (for example, the 17.05 to 16.95 ka time steps were used for 17 ka mean time slice).

### Establishing the presence and absence of prey animals

Two databases of dated faunal material were constructed to understand the movement of two key prey species, *Rangifer tarandus* (reindeer) and *E. ferus* (horse). These Upper Palaeolithic human prey species constitute the main population of cut marked bones used to assess human presence on the landscape of the British land mass. The *R. tarandus* data were extracted from Sommer et al.^76^ and updated using the Radiocarbon Palaeolithic database^100^. In both datasets, all radiocarbon-dated reindeer <15° E, >30° N and younger than 20,000 ^14^C years were included in the subsequent analysis. This included some radiocarbon-dated specimens that had dates only through association with other artefacts. While these contextual dates might weaken some chronological associations, they provide a broader set of data for comparison. Dates from the Radiocarbon Palaeolithic database were extracted from v.29 of the database accessed via https://ees.kuleuven.be/en/geography/projects/14c-palaeolithic/index.html. New dates were identified by using both the geographical and chronological constraints imposed on the Sommer et al. data^76^. Specimens referred to as containing either “*Rangifer*” or “Reindeer” were retained, and any duplicates with the Sommer data were identified and removed. The same exercise was completed for *E. ferus* using the criteria outlined above, but the data were only drawn from the Radiocarbon Palaeolithic database (v.29). This process identified 691 reindeer dates and a further 243 horse dates. The difference in numbers means more weight is placed on the outcome of the *R. tarandus* analysis than that for *E. ferus*.

**Extended Data Fig. 7:**
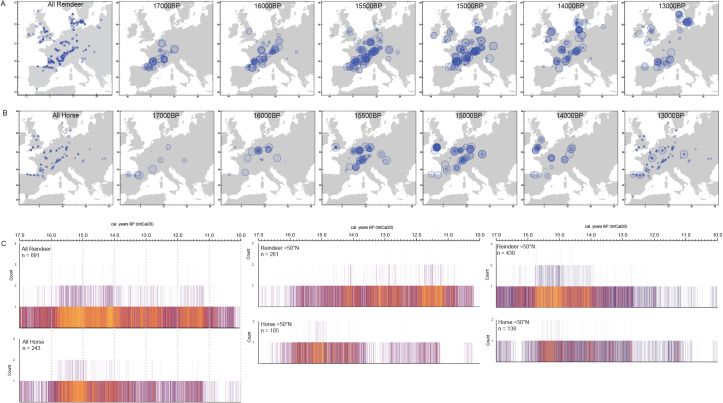
Faunal occurrence Data from across Europe for reindeer and horse. **A**): Changes in the distribution of *Rangifer tarandus* (reindeer) in NW Europe from 16.5 to 13.5 ka BP. The upper left panel shows all data included with specific time slices labelled in other panels. Circle size is related to probability with larger circles suggesting high probability of species presence. This demonstrates increased likelihood of *Rangifer tarandus* existing in Southern Britain after 16 ka and an expansion of the number of Rangifer locations between 15.5 – 15 ka. **B**) Changes in the distribution of *Equus ferus* in NW Europe from 17.0 to 13.0 ka BP. The upper left panel shows all data included with specific time slices labelled in other panels. Circle size is related to probability with larger circles suggesting high probability of species presence. This demonstrates high probabilities of *Equus ferus* existing in Southern Britain from 15.5 – 15 ka and continuous presence in Britain until c. 13 ka). **C**) Heat map for the timing of presence in the landscape of *Rangifer tarandus* (reindeer) and *Equus ferus* (horse) in NW Europe from 17.0 ka to 10 ka BP (For total distribution of dates see the upper left panels of supplementary figures 4.1 and 4.2. The panels show the presence of all horse and reindeer, and then the phasing of horse and reindeer above and below 50° N. RECEs are plotted based on the colourmap of Stewart et al.^13^ this states that brighter colours (Orange to yellows) represent higher density regions, while darker colours (purples) show and lower density regions. Absences of dated counts are left blank. The *y*-axis represents the count. For instance, for Reindeer >50°N a count of three is observed at ca 12.8 ka. This occurs when three dated events occur in the same year in an individual REC member.

**Extended Data Fig. 8:**
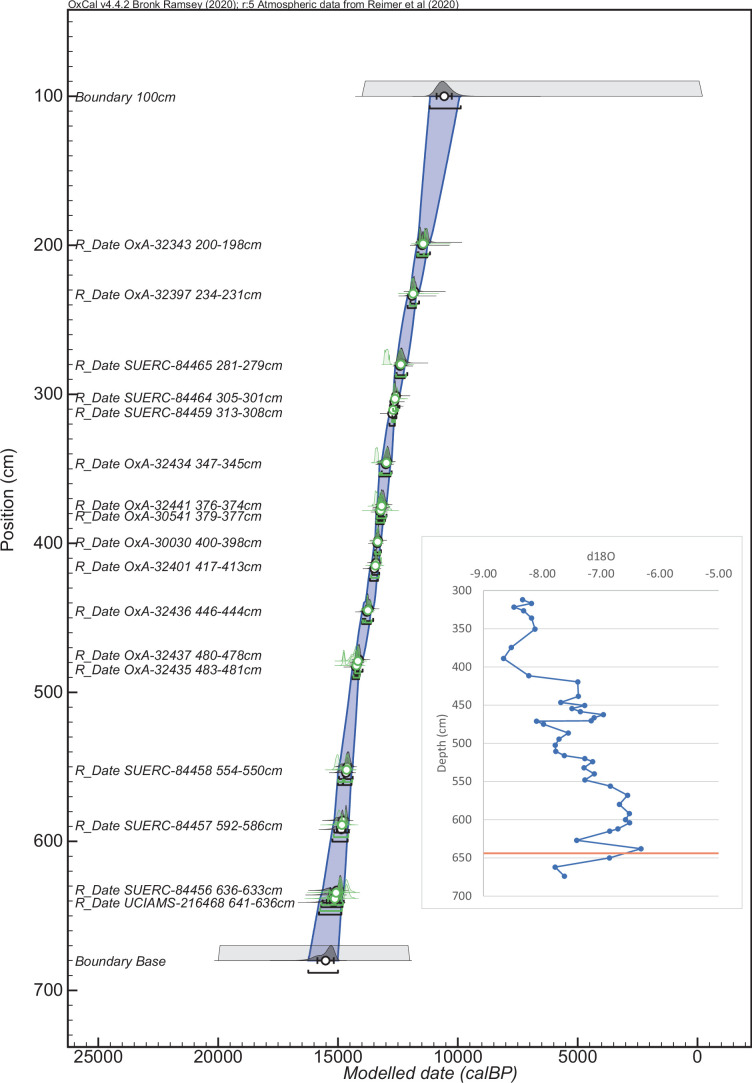
The revised age model for Wykeham. Revised age model for Wykeham. This age model is identical to that generated by Lincoln et al.^57^, but uses the updated IntCal20 to calibrate the dates. This recalibration was required to make the record directly comparable to the other data within this study. The inset figure shows δ^18^O content of the carbonate against sediment core depth at Wykeham as reported by Lincoln et al.^57^,. The conservative depth used to represent warming and then compared with temperature increases at Llangorse is 641 cm (marked by the orange horizontal line).

The total spatial range of the data and broadly mapped calibrated dates are provided as 1,000–500-year time slices in Extended Data Fig. 7 (calculated in OxCal v.4.4 and using the IntCal20 calibration curve^39,77^). The spatial analysis suggests that both *R. tarandus* and *E. ferus* expand their ranges into the NW European margin after 16 ka and are constantly present after 15.5 ka. However, these qualitative spatial analyses often fail to account for the chronological uncertainty and likelihood for preservation biases within the data alongside issues with the radiocarbon calibration curve leading to spurious associations^101^. Here we used a RECE approach as an attempt to circumvent some of these issues. RECE analysis provides a series of event count sequences, which are subsequently presented in an ensemble to reflect the probability of a count at a particular time step. It draws samples from the larger population of possible dates, optimizes these and presents them graphically^13^. The results of this analysis are presented as a heat map in Fig. 4 and Extended Data Fig. 7, with presence and absence identifiable with brighter colours reflecting increased probability of a count in a particular time step^101^. Where more than a single count is identified in a time step in a single model run, the *y* axis reflects this by placing a marker against two, three or more ‘counts’. This approach is thought far more robust than summing radiocarbon probabilities but may have the detrimental effect of extending distributions slightly earlier and later in time.

The results of the RECE were split into NW and southwest Europe using the 50° N line of latitude, as this separated the chosen archaeological sites into the >50° N grouping and used Llangorse as a guide for location. We recognize that this selection of 50° N might be considered arbitrary, but it follows the suggestion of Sommer et al., amalgamating their analytical zones 1–3 (British land mass, southern Scandinavia and northern central Europe)^76^. RECE analyses were then carried out on the whole dataset and the latitudinally separated data. These analyses demonstrate that both *R. tarandus* and *E. ferus* expanded their range into northern Europe >50° N after ~16 ka with a particularly strong association for horse between 15.5 and 15 ka.

### Reporting summary

Further information on research design is available in the Nature Portfolio Reporting Summary linked to this article.

## Supplementary information

Supplementary Information Supplementary Table 1: Date table including dated materials. Supplementary Section 1: Description of the choices behind analysis of the archaeological materials. Supplementary Section 2: Chironomid palaeoecological descriptions. Supplementary Section 3: Description of the drivers of the oxygen isotope signal. Supplementary Section 4: Determining the sea ice signal and choices for analysis.

Reporting Summary

## Data Availability

All raw data are available via figshare at 10.17637/rh.22133483.v1 (ref. ^102^) and 10.17637/rh.28043204.v1 (ref. ^103^).

## Code availability

The custom code generated for this project is available via figshare at 10.17637/rh.28052807.v1 (ref. ^104^), 10.17637/rh.28045037.v1 (ref. ^105^), 10.17637/rh.22133474.v1 (ref. ^106^) and 10.17637/rh.28043207.v1 (ref. ^107^). All other code generated by other authors is cited in the Methods.
