# Supplementary material for: Summer warmth between 15,500 and 15,000 years ago enabled human repopulation of the northwest European margin
Source: Nat Ecol Evol. 2025 Jul 2;9(7):1179–92. doi: 10.1038/s41559-025-02712-9 (PMC12240825; doi:10.1038/s41559-025-02712-9)
Supplement: Supplementary file 1 — Supplementary Table 1: Date table including dated materials. Supplementary Section 1: Description of the choices behind analysis of the archaeological materials. Supplementary Section 2: Chironomid palaeoecological descriptions. Supplementary Section 3: Description of the drivers of the oxygen isotope signal. Supplementary Section 4: Determining the sea ice signal and choices for analysis. [file 41559_2025_2712_MOESM1_ESM.pdf]

nature ecology & evolution

Supplementary information

https://doi.org/10.1038/s41559-025-02712-9

Summer warmth between 15,500 and 15,000 years ago enabled human repopulation of the northwest European margin

In the format provided by the authors and unedited

Supplementary Table 1. The radiocarbon dates obtained for Llangorse core LLAN14.

Publication Code	Sample identifier	^{14}C Enrichment	Conventional ^{14}C Age (1 sig)	δ^{13}C	Unmodelled Age	95.4% cal. BP range	Sampled material
UCIAMS-210605	LLAN 14 199-200	33.06 ± 0.38	8890 ± 100		9963<br>(9630-10235)	9782<br>(9532-10236)	3 x *Betula* sp. fruit; 1 x bud scale
SUERC-91940	LLAN 14 250-254	32.78 ± 0.17	8960 ± 41	-26.2	10089<br>(9911-10230)	10275<br>(9963-10807)	6 x seeds indeterminate c.f. Ranunculaceae; 1 x wood fragment; 2 x seeds indeterminate c.f. Ranunculaceae; 1 x bud scale
UCIAMS-210604	LLAN 14 279-281	28.87 ± 0.13	9980 ± 40		11447<br>(11265-11689)	10821<br>(10461-11131)	3 x *Betula* sp. Fruit
SUERC-81796	LLAN 14 303-305	29.36 ± 0.15	9845 ± 40	-27.6	11253<br>(11194-11389)	11245<br>(11190-11322)	10 x *Betula* tree fruits, 3 x *Betula* tree catkins, 8 x *Betula c.f. pendula* leaf fragments, 2 x *Populus tremula* catkins
UCIAMS-225393	LLAN 14 310-311	28.81± 0.33	10000 ± 100		11523<br>(11232-11829)	11359<br>(11235-11569)	3 x *Populus tremula* catkins, 1 x *Betula* sp. fruit c.f. *Betula* leaf fragments
UCIAMS-210595	LLAN 14 319-321	27.99 ± 0.10	10230 ± 30		11900<br>(11765-12000)	11489<br>(11288-11858)	6 x *Betula* fruits, 3 x *Betula* catkins, *Betula* leaf fragments, 2 x *Populus tremula* catkins, 1 x *Juniperus communis* needle, 1 x bud scale indeterminate.
UCIAMS-229591	LLAN 14 327-329	27.92 ± 0.24	10250 ± 70		12017<br>(11655-12465)	11579<br>(11369-11896)	1 x *Betula* catkin scale tree type. 3 x wood indeterminate.
UCIAMS-210596	LLAN-14 348-352	29.10 ± 0.11	9915 ± 30		11316<br>(11240-11399)	11715<br>(11480-11981)	3 x *Betula nana* fruits; 24 x leaf fragments indeterminate, 4 x bud scales indeterminate, 3 x c.f. *Betula* twigs

UCIAMS-229592	LLAN 14 352-356	27.92 ± 0.10	10250 ± 30		11935 (11820-12093)	11774 (11553-12039)	9 x *Betula nana* fruit, > 10 *Betula nana* bud scales, twigs indeterminate, leaf fragments c.f. *Betula nana*. 1 x ericaceous seed, 3 x *Betula nana* bud scale, leaf fragments c.f. *Betula*, twigs indeterminate.
UCIAMS-229593	LLAN 14 366-370	28.37 ± 0.12	10120 ± 35		11714 (11405-11928)	11862 (11648-12108)	2 x *Betula nana* leaf and many *Betula nana* leaf fragments, 6 x *Betula nana* fruit, 1 x *Salix herbacea*, 1 x ericaceous leaf and many leaf fragments indeterminate, 4 x *Betula* bud scales c.f. *Betula nana*. 1 x *Betula nana* catkin, twigs indeterminate, 1 x seed (indeterminate)
UCIAMS-229594	LLAN 14 402-408	29.18 ± 0.10	9895 ± 30		11295 (11233-11395)	12114 (11893-12343)	3 x *Betula* bud scales; 2 x *Betula nana* leaf 1 x *Betula* male catkin scale, wood fragments, leaf fragments. 1 x *Betula* leaf, 1 x *Betula* fruit tree type. 1 x ericaceous seed, leaf fragments c.f. *Betula*
UCIAMS-229595	LLAN14 480-484	26.98 ± 0.12	10525 ± 40		12544 (12472-12678)	12626 (12483-12711)	1 x *Silene/Lychnis* seed; 1 x ericaceous leaf; 1 x Lenticular *Carex* seed; 1 x Poaceae seed; 1 x *Betula nana* fruit, 1 x *Betula nana* budscale; leaf fragments cf *Betula*, wood fragments (indeterminate); twigs; *Rumex acetosa*,
UCIAMS-229596	LLAN14 488-492	25.55 ± 0.28	10960 ± 90		12901 (12752-13075)	12815 (12734-12915)	twig ericaceous, ericaceous leaf *Empetrum*, leaf cf. *Betula*, *Rumex acetosa* seed, leaf fragments, *Betula* bud scale (nana-type); charred wood fragments, *Betula* leaf fragments, *Juncus* seed, woody fragments (indeterminate) Poaceae seed, *Silene/Lychnis* type seed.

UCIAMS-210603	LLAN 14 501-502	25.04 ± 0.09	11125 ± 35		13044 (12925-13111)	12949 (12843-13037)	1 x c.f. *Betula* sp. twig, 4 x leaf fragments, 2 x *Saxifraga aizoides* - seeds.
UCIAMS-210597	LLAN 14 508-511	24.13 ± 0.10	11420 ± 35		13283 (13178-13404)	13003 (12916-13192)	10 x *Betula* tree fruits, 1 x small c.f. *Betula* twig, *Betula* sp. Leaf fragments, 1 x *Saxifraga aizoides* seed, 1 x Asteraceae seed
UCIAMS-225394	LLAN 14 515-516	25.61 ± 0.30	10940 ± 100		12891 (12742-13076)	13059 (12971-13157)	4 x *Betula* sp. fruits, 1 x c.f. *Betula* sp. bud scale, undifferentiated leaf fragments.
UCIAMS-225395	LLAN 14 525-526	24.69 ± 0.20	11240 ± 70		13154 (13010-13376)	13161 (13090-13244)	4 x *Betula* sp. fruits, 1 x *Betula* catkin and *Betula* leaf fragments.
UCIAMS-210602	LLAN-14 534-535	24.22 ± 0.12	11390 ± 40		13257 (13171-13334)	13262 (13180-13326)	3 x *Betula* sp. fruits, 14 x leaf fragment, 1 x twig.
UCIAMS-229597	LLAN 14 535-538	33.7 ± 0.12	8735 ± 30		9697 (9552-9889)	13326 (13218-13435)	2 x *Betula* leaf fragment; 1x *Betula* budscale; 6 x *Betula* sp. fruit; 1 x *Salix* budscale; 1 x Poaceae seed; 1 x *Carex* seed; 1 *Betula* tree-type fruit; twigs indeterminate; leaf indeterminate;
SUERC-81797	LLAN 14 548-549	23.54 ± 0.13	11619 ± 45	-28.1	13483 (13353-13589)	13478 (13365-13584)	3 x *Betula* tree fruits, 3 x *Betula* sp. twigs, 1 x *Betula* sp. catkin scale, bark.
SUERC-82300	LLAN 14 565-566	21.61 ± 0.13	12306 ± 47	-27.8	14331 (14083-14810)	13670 (13511-13885)	13 x *Betula* sp. fruits, leaf fragments x 5, 1 x female catkin, 1 x *Taraxacum* sp. seed, 1 x twig undiff.
UCIAMS-229599	LLAN 14 573-574	22.98 ± 0.18	11810 ± 70		13668 (13505-13796)	13784 (13624-14012)	7 x *Betula* tree-type fruit; 2 x *Betula* nana fruit; 2 x *Betula* sp. bud scale; 1 x Poaceae; 1 x Ranunculaceae seed

UCIAMS-225396	LLAN 14 582-584	21.96 ± 0.09	12180 ± 35		14085 (13886-14185)	14076 (14010-14170)	7 x *Betula* tree fruits, 3 x *Betula* nana fruits, *Betula* sp. Leaf fragments, 1 x *Rumex acetosella* seed.
UCIAMS-229600	LLAN 14 590-591	21.63 ± 0.17	12300 ± 70		14360 (14060-14828)	14269 (14088-14504)	2 x *Betula nana* leaf fragment
UCIAMS-225397	LLAN 14 606-610	20.98 ± 0.12	12545± 45		14854 (14523-15115)	14693 (14298-15061)	1 x *Betula nana* fruit, 1 x *Betula* nana catkin, 3 x *Dryas octopetala* leaves, leaf fragments undifferentiated.
UCIAMS-210598	LLAN 14 610-614	20.44 ± 0.18	12750 ± 80		15216 (14977-15498)	14839 (14339-15305)	1 x *Betula nana* fruit, leaf fragments (ericaceous and c.f. *Betula*), c.f. *Betula* bud scales.
UCIAMS-274874	LLAN21 653-655	21.19 ± 0.11	12463 ± 41		14650 (14322-14964)	15285 (14655-16108)	1 x twig indeterminate

Supplementary information 1. Recalibration of radiocarbon dates and age modelling of material from British Upper Palaeolithic sites

The radiocarbon data presented by Jacobi and Higham (2011) were remodelled in OxCal v4.4 utilising the IntCal20 calibration curve. Each site was included in an OxCal 'Phase' model and the likely onset of each phase extracted for comparison with the Llangorse data. The approach mirrors that of the original study with some minor differences in individual treatment of grouping certain dates. These modifications are outlined below. Initially it was intended that each site would be processed exactly as the original study however, some divergence occurred in certain sites when pre-processing data. No differences in data selection are present for King Arthur's Cave or Kents Cavern and these are not discussed further.At Sun Hole, radiocarbon samples OxA-14476 and OxA-14477 were from the same individual and therefore were combined (R_Combine) prior to construction of the phase, here they are referred to as '*Equus ferus* dent'. At Gough's Cave, sample OxA-18067 was included in the analysis as it was detected alongside the other dated individuals. Jacobi and Higham considered this to be an example of later human activity at the site and do not include it. The Phase was carried out including and excluding this material. The results of this test show that in- or excluding the material does not alter the onset of the timing of activity in the phase so the date was left in for completeness. At the various Cresswell locations, the data presented here differs from Jacobi and Higham's analysis by being split by location and not species. Jacobi and Higham split the dates into either *Equus ferus* or *Lepus timidus* groups based on a test of which was oldest. However, as they concluded that no clear chronological offset can be observed with the available data the data here have been combined per excavation site instead of per organism.

Supplementary information 2. Llangorse Chironomid Analysis:

Palaeoecological description

Here we include a description of the chironomid fauna in the zones presented in Extended Data Figure 3.

Zone 1.

During zone 1 (15,477-15,206 cal. yr BP), ultra-cold and cold indicating taxa dominated the assemblages including *M. radialis*-type (*c.* 20 %), *Paracladius* (*c.* 20 %) and COTL-type (*c.* 60%). Warm indicating taxa are largely absent suggesting the prevalence of very cold climate

conditions. The taxa present also suggest nutrient poor conditions (Walker et al 1991; Brodin 1986; Brooks et al., 2007). *Paracladius* requires well oxygenated water with saturation over 50 % day and night (Moller Pillot, 2013).

Zone 2

In zone 2 (15,206-12,901 cal. yr BP), a notable shift in the assemblage composition occurs with cold indicating taxa decreasing to only a few percent while cool-temperate, temperate and warm indicating taxa display a large increase such as *Psectrocladius sordidellus*-type (*c.* 20 %), *Ablabesmyia (c.*15 %), and *Tanytarsus glabrescens*-type (*c.* 10 %). Other warm adapted taxa are also consistently present at lower abundances, *c.* 1-5 % including *Nanocladius rectinervis*-type, *Nanocladius branchicolus*-type, and *Parachironomous varus*-type. The assemblages are composed of taxa suited to a range of nutrient levels such as the eutrophic indicating taxa *T. glabrescens*-type (Brodin 1986; Bilyj and Davies, 1989), *E. pagana*-type (Kansanen 1985; Saether, 1979) and *Nanocladius* (Moller Pillot, 2013; Cranston et al., 1983) along with mesotrophic indicating taxa such as *P. sordidellus*-type (Moller Pillot, 2013) suggesting the lake is meso-eutrophic. The lake is likely to have been reasonably well oxygenated as indicated by the presence of *Microtendipes pedellus*-type (Steenbergen, 1993).

Zone 3

For zone 3 (12,901-11,718 cal. yr BP), cool temperate, temperate, and warm taxa decline but continue to be present in low abundances including *Psectrocladius sordidellus*-type (*c.* 10 %), *Ablabesmyia (c.* 5 %), and *Tanytarsus glabrescens*-type (*c.* 1 %). Ultra-cold and cold indicating taxa increase and form a notable portion of the assemblage including *Paracladius* (*c.* 20 %), *Heterotrissocladius grimshawi*-type (*c.* 20 %) and *Parakieferiella nigra*-type (*c.* 5 %). Taxa suited to a range of different nutrient levels, e.g., *Heterotrissocladius grimshawi*-type (oligotrophic; Saether 1979), *P. sordidellus*-type (mesotrophic; Moller Pillot, 2013) and *T. glabrescens*-type (eutrophic; Brodin 1986) are all present. Overall, the lake is likely to be oligo-mesotrophic. Lake waters are well oxygenated as required by *H. grimshawi*-type, *Paracladius* and *M. pedellus*-type (Moller-Pillot, 2013; Steenbergen et al., 1993).

Zone 4

During zone 4 (11,718-10,611 cal. yr BP), cool-temperate, temperate, and warm-indicating taxa increase again and dominate the assemblage such as *Ablabesmyia (c.* 30 %), *Dicrotendipes* spp. (*c.* 15 %) and *Tanytarsus glabrescens*-type (*c.* 10 %). Ultra-cold and cold indicating taxa once again largely disappear. As in the Lateglacial Interstadial, the assemblages are composed of taxa suited to a range of nutrient levels e.g., *T. glabrescens*-type (Brodin 1986; Bilyj and Davies, 1989), *Nanocladius* (Moller Pillot, 2013; Cranston et al., 1983) and *P. sordidellus*-type (Moller Pillot, 2013) suggesting the lake is meso-eutrophic. The lake continues to be reasonably well oxygenated as indicated by the presence of *Microtendipes pedellus*-type, which requires well oxygenated water (Brooks et al 2007; Steenbergen, 1993).

Supplementary information 3. What drives the Llangorse stable isotope signal?

Interpretation of the $\delta^{18}O$ values of any carbonate sequence is complicated by the fact that this value is controlled by two variables neither of which can be reliably quantified in the geological past (see Leng and Marshall, 2004 for discussion). These two variables are the; 1) the fractionation of isotopes that occurs during the mineralisation of the carbonate, which is controlled by the prevailing temperature, and 2) the $\delta^{18}O$ value of the water from which the carbonate mineralises. The fractionation of isotopes during mineralisation is well-defined through empirical studies and approximates to ca -0.25 to -0.30‰ per +1°C (Hays and Grossman, 1991; Kim and O'Neill, 1997). This means that, if the $\delta^{18}O$ value of the source water remains unchanged, then carbonates that precipitate under higher temperatures will have lower $\delta^{18}O$ values than those that precipitate under higher temperatures. In Lateglacial and early Holocene lake carbonate sequences, $\delta^{18}O$ values, when compared to C-IT_{Jul} estimates from the same sequence (Marshall et al., 2002; Van Asch et al., 2012; Blockley et al., 2018), do not follow this pattern with higher $\delta^{18}O$ values occurring in association with higher temperature reconstructions and vice versa. This is also true for the Llangorse sequence. Consequently, it is widely assumed that the major driver of Lateglacial $\delta^{18}O$ records in Northwest Europe is changes in the $\delta^{18}O$ of the lake water, however, this value can, in turn be controlled by multiple factors.

First of these is the $\delta^{18}O$ value of rainfall, which is the ultimate source of the lake water (Rozanski et al., 1992; 1993; Darling and Talbot, 2003). Whilst a range of factors may control the $\delta^{18}O$ value of rainfall most of this cause a positive linear relationship to exist between air temperatures and the $\delta^{18}O$ of rainfall (at a quantified relationship of ~+0.58‰ per +1°C, see Rozanski et al., 1992; 1993). Air temperature controls the fractionation of oxygen isotopes as precipitation condenses and falls, with warmer temperatures producing more positive $\delta^{18}O$

values. Air mass source/trajectory is also influential with, in the British Isles, rainfall sourced from the south and west characterised by more positive $\delta^{18}O$ values than that sourced from more northerly latitudes (Darling and Talbot, 2003). As more northerly air masses are also associated with colder air temperatures than more southerly air masses this enhances the positive relationship between air temperature and rainfall $\delta^{18}O$ values. Finally, the amount of rainfall is also significant in controlling the $\delta^{18}O$ value of rainfall (Darling and Talbot, 2003), however, as colder air frequently carries less moisture than warmer air this also leads to a relationship existing between air temperature and rainfall $\delta^{18}O$ values. All these factors typically produce more positive $\delta^{18}O$ values in rainfall under increasing temperatures.

Environmental processes that operate during the movement of rainfall through surface and sub-surface flow to recharge the lake basin may result in the homogenisation of the $\delta^{18}O$ of water resulting in less seasonal differences in groundwater/river waters than in the source rainfall (Darling et al., 2003). Finally, processes such as evaporation can modify the $\delta^{18}O$ value of lake waters at a rate controlled by the aridity of the climate and the residence time/recharge rate of the lake basin (Talbot, 1990; Darling et al., 2003; Leng and Marshall, 2004). The clearest evidence for a strong degree of isotopic modification in a lake basin is through co-variance between $\delta^{18}O$ and $\delta^{13}C$ values, as increasing rates of evaporation will cause both isotopic variables to increase due to both the direct effect of evaporation and the effect of evaporation on the volume of the water body (Talbot, 1990). As significant evidence only exists for $\delta^{18}O$ and $\delta^{13}C$ co-variance in the section of Llangorse that is found at the very end of the Lateglacial interstadial, see SI Figure 2.3, (13.51 to 12.74 cal. ka BP) inter-basin evaporitic modification of the $\delta^{18}O$ value of lake water is not characteristic of Llangorse lake for most of the Lateglacial interstadial and the early Holocene. The increase in evaporitic enrichment that is seen at the very end of the interstadial is characteristic of the trend towards drier climates and falling lake levels, resulting in a smaller lake volume and, consequently, greater susceptibility to evaporitic modification, that is seen as the onset of the Lateglacial stadial approaches (Lincoln et al., 2020).

In summary, we use the evidence from the Llangorse sequence to argue that the following two assumptions are valid. Firstly, that the $\delta^{18}O$ values of the lake carbonates are primarily controlled by changes in the $\delta^{18}O$ values of the lake water, which, with the exception of the section from 13.51 to 12.74 cal. ka BP (Zone 3) where there is evidence for $\delta^{18}O$ and $\delta^{13}C$ co-variance, is, in turn, a function of changes in the $\delta^{18}O$ value of precipitation. Secondly, that changes in the $\delta^{18}O$ of precipitation, across this interval, are associated with changes in air temperatures, either through the direct effect of air temperature on the fractionation of isotopes

during condensation/rainfall or through changes between the source and trajectory of cold and warm air masses. This suggestion is supported by the relationship between $\delta^{18}O$ values in the Llangorse carbonates and the reconstructed C-IT_{Jul}. As such the increase in $\delta^{18}O$ values at the onset of the interstadial are suggested to reflect an increase in air temperature and is, therefore, a second line of evidence that suggest the onset of warm interstadial climates from at least 15.20 cal. ka BP.

Supplementary information 4. Sea Ice analysis and figures

The figures presented in Figure 1B and Figure 4 use estimate of palaeo-sea level and ice limits extracted from existing literature. Palaeoshorelines were generated from the CHELSA_Trace21ka model and utilised the code and methods of (Karger et al., 2023).

Karger, D.N., Nobis, M.P., Normand, S., Graham, C.H. and Zimmermann, N.E., 2023. CHELSA-TraCE21k–high-resolution (1 km) downscaled transient temperature and precipitation data since the Last Glacial Maximum. Climate of the Past, 19(2), pp.439-456.

Eurasian ice extenet was generated in millennial time slices and provided by Hughes et al. 2016.

Hughes, A.L., Gyllencreutz, R., Lohne, Ø.S., Mangerud, J. and Svendsen, J.I., 2016. The last Eurasian ice sheets–a chronological database and time-slice reconstruction, DATED-1. *Boreas*, *45*(1), pp.1-45

Last Glacial Maximum shapefiles for Greenland and continental Europe are from:

Ehlers, J., Gibbard, P.L. and Hughes, P.D. eds., 2011. *Quaternary glaciations-extent and chronology: a closer look* (Vol. 15). Elsevier.

Defining the 'sea ice fraction'

Simulations of sea-ice fraction (ICEFRAC) and surface temperature (TS) were obtained from the transient global, coupled ocean-atmosphere-sea ice- land surface climate model simulation TraCE-21ka (Liu et al., 2009; He, 2011 https://www.earthsystemgrid.org/project/trace.html). The sea-ice model employed in TraCE 21ka is the dynamic-thermodynamic National Center for Atmospheric Research (NCAR) Community Sea Ice Model (CSIM) with a longitudinal resolution of 3.6^{o} and a variable latitudinal resolution, with finer resolution near the equator (~0.9^{o}). CSIM considers both the dynamic (ice movement and deformation) and thermodynamic (heat transfer and phase changes) processes that influence sea-ice extent and thickness. The model also includes subgrid-scale ice thickness distributions to account for complex small-scale variability in ice extent (Briegleb et al., 2004; He, 2011). This makes simulated sea-ice coverage sensitive to atmospheric and oceanic variability, as well as the internal ice properties (Briegleb et al., 2004; Otto-Bliesner et al., 2006).

Reconstructions of sea-ice extent were made using the ICEFRAC variable from the transient TraCE-21ka model simulation, which represents the proportion of a grid cell's surface area covered by sea-ice. The variable ranges between 0 to 1, where 0 indicates no sea-ice coverage in the grid cell, and 1 indicates complete coverage. The ICEFRAC variable can therefore be used to quantify the changing extent of sea-ice in the North Atlantic and Nordic Seas in response to palaeoclimatic change.

References.

Bilyj, B. and Davies, I.J., 1989. Descriptions and ecological notes on seven new species of Cladotanytarsus (Chironomidae: Diptera) collected from an experimentally acidified lake. Canadian Journal of Zoology, 67(4), pp.948-962.

Blockley, S., Candy, I., Matthews, I., Langdon, P., Langdon, C., Palmer, A., Lincoln, P., Abrook, A., Taylor, B., Conneller, C. and Bayliss, A., 2018. The resilience of postglacial hunter-gatherers to abrupt climate change. Nature ecology & evolution, 2(5), pp.810-818.

Briegleb, B.P., Bitz, C.M., Hunke, E.C., Lipscomb, W.H., Holland, M.M., Schramm, J.L. and Moritz, A.R., 2004. Scientific description of the sea ice component in the Community Climate System Model. Version, 3, p.70

Brodin, Y. W. (1986). The postglacial history of Lake Flarken, Southern Sweden, interpreted from subfossil insect remains. Hydrobiology, 71(3), 371-432.

Brooks, S. J, Langdon. P. G, Heiri, O. 2007. The identification and use of Palaearctic Chironomidae larvae in palaeoecology. *Quaternary Research Association.* Technical Guide No.10.

Cranston, D.R., 1983. The larvae of Orthocladiinae (Diptera, Chironomidae) of the Holarctic region-Keys and diagnoses. Entomologica Scandinavica, Supplement, 19, pp.149-291.

Darling, W.G. and Talbot, J.C., 2003. The O and H stable isotope composition of freshwaters in the British Isles. 1. Rainfall. Hydrology and Earth System Sciences, 7(2), pp.163-181.

Darling, W.G., Bath, A.H. and Talbot, J.C., 2003. The O and H stable isotope composition of freshwaters in the British Isles. 2. Surface waters and groundwater. Hydrology and Earth System Sciences, 7(2), pp.183-195.

Hays, P.D. and Grossman, E.L., 1991. Oxygen isotopes in meteoric calcite cements as indicators of continental paleoclimate. Geology, 19(5), pp.441-444.

He, F.: Simulating Transient Climate Evolution of the Last deglaciation with CCSM3, Doctor of Philosophy, Atmospheric and Oceanic Sciences, University of Wisconsin-Madison, Wi, USA, 161 pp., 2011.

Kansanen, P.H., 1985, January. Assessment of pollution history from recent sediments in Lake Vanajavesi, southern Finland. II. Changes in the Chironomidae, Chaoboridae and Ceratopogonidae (Diptera) fauna. In Annales Zoologici Fennici (pp. 57-90). Finnish Academy of Sciences, Societas Scientiarum Fennica, Societas pro Fauna et Flora Fennica and Societas Biologica Fennica Vanamo.

Kim, S.T. and O'Neil, J.R., 1997. Equilibrium and nonequilibrium oxygen isotope effects in synthetic carbonates. Geochimica et cosmochimica acta, 61(16), pp.3461-3475.

Leng, M.J. and Marshall, J.D., 2004. Palaeoclimate interpretation of stable isotope data from lake sediment archives. Quaternary Science Reviews, 23(7-8), pp.811-831.

Lincoln, P.C., Matthews, I.P., Palmer, A.P., Blockley, S.P., Staff, R.A. and Candy, I., 2020. Hydroclimatic changes in the British Isles through the Last-Glacial-Interglacial Transition: multiproxy reconstructions from the Vale of Pickering, NE England. Quaternary Science Reviews, 249, p.106630.

Liu, Z., Otto-Bliesner, B.L., He, F., Brady, E.C., Tomas, R., Clark, P. U., Carlson, A. E., Lynch-Stieglitz, J., Curry, W., Brook, E., Erickson, D., Jacob, R., Kutzbach, J., Cheng J. 2009. Transient simulation of last deglaciation with a new mechanism for Bølling-Allerød warming. *Science* **325**, 310–314.

Marshall, J.D., Jones, R.T., Crowley, S.F., Oldfield, F., Nash, S. and Bedford, A., 2002. A high resolution late-glacial isotopic record from Hawes Water, northwest England: Climatic oscillations: Calibration and comparison of palaeotemperature proxies. Palaeogeography, Palaeoclimatology, Palaeoecology, 185(1-2), pp.25-40.

Moller Pillot, H. (2013). Chironomidae Larvae, Vol. 3: Orthocladiinae. Leiden, The Netherlands: KNNV Publishing.

Otto-Bliesner, B.L., Brady, E.C., Clauzet, G., Tomas, R., Levis, S. and Kothavala, Z., 2006. Last glacial maximum and Holocene climate in CCSM3. Journal of Climate, 19(11), pp.2526 2544.

Rozanski, K., Araguas-Araguas, L. and Gonfiantini, R., 1992. Relation between long-term trends of oxygen-18 isotope composition of precipitation and climate. Science, 258(5084), pp.981-985.

Rozanski, K., Araguás-Araguás, L. and Gonfiantini, R., 1993. Isotopic patterns in modern global precipitation. Climate change in continental isotopic records, 78, pp.1-36.

Saether, O.A., 1979. Chironomid communities as water quality indicators. Ecography, 2(2), pp.65-74.

Steenbergen, C.L.M., Sweerts, J.P. and Cappenberg, T.E., 1993. Microbial biogeochemical activities in lakes: stratification and eutrophication. 4. Aquatic Microbiology. An Ecological Approach.

Talbot, M.R., 1990. A review of the palaeohydrological interpretation of carbon and oxygen isotopic ratios in primary lacustrine carbonates. Chemical Geology: Isotope Geoscience Section, 80(4), pp.261-279.

van Asch, N., Lutz, A.F., Duijkers, M.C., Heiri, O., Brooks, S.J. and Hoek, W.Z., 2012. Rapid climate change during the Weichselian Lateglacial in Ireland: Chironomid-inferred summer temperatures from Fiddaun, Co. Galway. Palaeogeography, Palaeoclimatology, Palaeoecology, 315, pp.1-11.

Walker, I.R., Mott, R.J. and Smol, J.P., 1991. Allerød—Younger Dryas lake temperatures from midge fossils in Atlantic Canada. Science, 253(5023), pp.1010-1012.
