# Supplementary material for: Summer warmth between 15,500 and 15,000 years ago enabled human repopulation of the northwest European margin
Source: Nat Ecol Evol. 2025 Jul 2;9(7):1179–92. doi: 10.1038/s41559-025-02712-9 (PMC12240825; doi:10.1038/s41559-025-02712-9)
Supplement: Supplementary file 2 — Reporting Summary [file 41559_2025_2712_MOESM2_ESM.pdf]

nature portfolio

Corresponding author(s): Ian Matthews i.p.matthews@rhul.ac.uk and Adrian Palmer a.palmer@rhul.ac.uk

Last updated by author(s): Dec 17, 2024

Reporting Summary

Nature Portfolio wishes to improve the reproducibility of the work that we publish. This form provides structure for consistency and transparency in reporting. For further information on Nature Portfolio policies, see our Editorial Policies and the Editorial Policy Checklist.

Statistics

For all statistical analyses, confirm that the following items are present in the figure legend, table legend, main text, or Methods section.

n/a	Confirmed	
☐	☒	The exact sample size (*n*) for each experimental group/condition, given as a discrete number and unit of measurement
☐	☒	A statement on whether measurements were taken from distinct samples or whether the same sample was measured repeatedly
☐	☒	The statistical test(s) used AND whether they are one- or two-sided<br>*Only common tests should be described solely by name; describe more complex techniques in the Methods section.*
☐	☒	A description of all covariates tested
☐	☒	A description of any assumptions or corrections, such as tests of normality and adjustment for multiple comparisons
☐	☒	A full description of the statistical parameters including central tendency (e.g. means) or other basic estimates (e.g. regression coefficient) AND variation (e.g. standard deviation) or associated estimates of uncertainty (e.g. confidence intervals)
☐	☒	For null hypothesis testing, the test statistic (e.g. *F*, *t*, *r*) with confidence intervals, effect sizes, degrees of freedom and *P* value noted<br>*Give P values as exact values whenever suitable.*
☐	☒	For Bayesian analysis, information on the choice of priors and Markov chain Monte Carlo settings
☐	☒	For hierarchical and complex designs, identification of the appropriate level for tests and full reporting of outcomes
☐	☒	Estimates of effect sizes (e.g. Cohen's *d*, Pearson's *r*), indicating how they were calculated

Our web collection on statistics for biologists contains articles on many of the points above.

Software and code

Policy information about availability of computer code

Data collection	Data collection did not use any specialised code or software.
Data analysis	Analysis of radiocarbon dated material was carried out using Oxcal version 4.4 https://c14.arch.ox.ac.uk/oxcal.html . The code used to generate the analyses has been lodged in our institutional figshare location and the link to this has been provided in the manuscript for the data and code availability statements. Translating the GICC05 timescale to cal. BP was undertaken in r 4.3.1 and the code is also provided by figshare. Changepoint analysis to define warming was undertaken using PAST v4.15 https://www.nhm.uio.no/english/research/resources/past/ . Plotting of chironomid and pollen data utilised c2 v1.8 https://www.staff.ncl.ac.uk/stephen.juggins/software/C2Home.htm . R packages 'Analogue' and 'vegan' were used to derive modern analogue information for the chironomid data using the standard approaches described in those packages documentation. Radiocarbon count ensembles of the faunal data were generated in R using the code of Stewart et al.2021 and the data for this analysis is logged with figshare.

For manuscripts utilizing custom algorithms or software that are central to the research but not yet described in published literature, software must be made available to editors and reviewers. We strongly encourage code deposition in a community repository (e.g. GitHub). See the Nature Portfolio guidelines for submitting code & software for further information.

Data

Policy information about availability of data

All manuscripts must include a data availability statement. This statement should provide the following information, where applicable:

- Accession codes, unique identifiers, or web links for publicly available datasets
- A description of any restrictions on data availability
- For clinical datasets or third party data, please ensure that the statement adheres to our policy

All raw data has been made available via our institutional repository hosted within figshare, available at: https://royalholloway.figshare.com/account/home#/projects/159914

Research involving human participants, their data, or biological material

Policy information about studies with human participants or human data. See also policy information about sex, gender (identity/presentation), and sexual orientation and race, ethnicity and racism.

Reporting on sex and gender	na.
Reporting on race, ethnicity, or other socially relevant groupings	na.
Population characteristics	na.
Recruitment	na.
Ethics oversight	na.

Note that full information on the approval of the study protocol must also be provided in the manuscript.

Field-specific reporting

Please select the one below that is the best fit for your research. If you are not sure, read the appropriate sections before making your selection.

☐ Life sciences ☐ Behavioural & social sciences ☒ Ecological, evolutionary & environmental sciences

For a reference copy of the document with all sections, see nature.com/documents/nr-reporting-summary-flat.pdf

Ecological, evolutionary & environmental sciences study design

All studies must disclose on these points even when the disclosure is negative.

Study description	This is a palaeoenvironmental study with aspects of palaeoecology, archaeology and chronology. The study uses sub-fossil remains from a lake sediment core to extract quantitative and qualitative palaeoenvironmental information. The study also utilises radiocarbon dating and the measurement of stable isotopes to look at the timing and significance of warming in the NW European margin for the timing of human repopulation
Research sample	This study combines primary data collected from sediment cores from Llangorse. From these samples, bulk sedimentological information was derived as was palaeoecological data from chironomids, pollen and plant remains. These plant remains were used for determining a radiocarbon chronology for the site. Stable isotope measurements were also collected from the sediments. Secondary data in the form of archaeological and faunal radiocarbon data were used to determine human presence and absence alongside occurrence of fauna.
Sampling strategy	The samples were taken at sufficient resolution to determine changing climatic conditions but not to overreach the potential of sediment sequences. Radiocarbon dates were obtained where samples permitted this and where sufficient dates were able to offer the required precision of the timing of climatic transitions.
Data collection	Data was collected by all authors from field sampling and subsequent laboratory sampling in the department of Geography at Royal Holloway
Timing and spatial scale	Samples were initially collected in July 2014, the last analyses (a radiocarbon date) was collected in 2023.
Data exclusions	No data were excluded.
Reproducibility	The core stratigraphy was repeated across several coring campaigns, but formal replicates of data were not sampled. This is common for this type of research.

Randomization	This is not relevent for our study as data could not be randomised from this type of palaeoenvironmental work.
Blinding	Blinding was not relevant this type of palaeoenvironmental work.

Did the study involve field work? ☒ Yes ☐ No

Field work, collection and transport

Field conditions	Fieldwork was carried out in a series of coring campaigns beginning in 2014.
Location	Sampling was undertaken using a Russian-type corer at the infilled margins of Llangorse 51.9357N, -3.2735W.
Access & import/export	Samples were retained and stored at 4 degrees in the RHUL cold store.
Disturbance	The sample hole (10 cm diameter) was carefully sealed after coring and all materials taken to site were removed.

Reporting for specific materials, systems and methods

We require information from authors about some types of materials, experimental systems and methods used in many studies. Here, indicate whether each material, system or method listed is relevant to your study. If you are not sure if a list item applies to your research, read the appropriate section before selecting a response.

Materials & experimental systems

n/a	Involved in the study
☒	☐ Antibodies
☒	☐ Eukaryotic cell lines
☐	☒ Palaeontology and archaeology
☒	☐ Animals and other organisms
☒	☐ Clinical data
☒	☐ Dual use research of concern
☒	☐ Plants

Methods

n/a	Involved in the study
☒	☐ ChIP-seq
☒	☐ Flow cytometry
☒	☐ MRI-based neuroimaging

Palaeontology and Archaeology

Specimen provenance	Only pre-existing aracheological data were used.
Specimen deposition	No specimens were access in the study, only secondary data.
Dating methods	New dates were obtained for the Llangorse sediment sequence. These are reported in full in the supplementry information and provided in spreadsheet format on figshare.

☒ Tick this box to confirm that the raw and calibrated dates are available in the paper or in Supplementary Information.

Ethics oversight	no ethical approval was required.

Note that full information on the approval of the study protocol must also be provided in the manuscript.

Plants

Seed stocks	na
Novel plant genotypes	na
Authentication	na
